# The Intragenesis and Synthetic Biology Approach towards Accelerating Genetic Gains on Strawberry: Development of New Tools to Improve Fruit Quality and Resistance to Pathogens

**DOI:** 10.3390/plants11010057

**Published:** 2021-12-25

**Authors:** Victoria Súnico, José Javier Higuera, Francisco J. Molina-Hidalgo, Rosario Blanco-Portales, Enriqueta Moyano, Antonio Rodríguez-Franco, Juan Muñoz-Blanco, José L. Caballero

**Affiliations:** Departamento Bioquímica y Biología Molecular, Campus de Rabanales, Edif. Severo Ochoa C6, Universidad de Córdoba, 14071 Cordoba, Spain; b12susam@uco.es (V.S.); b92hisoj@uco.es (J.J.H.); b52mohif@uco.es (F.J.M.-H.); bb2prblr@uco.es (R.B.-P.); bb2mocae@uco.es (E.M.); bb1rofra@uco.es (A.R.-F.); bb1mublj@uco.es (J.M.-B.)

**Keywords:** intragenic strawberry, synthetic biology, RNA interference, strawberry promoters, plant immunity

## Abstract

Under climate change, the spread of pests and pathogens into new environments has a dramatic effect on crop protection control. Strawberry (*Fragaria* spp.) is one the most profitable crops of the Rosaceae family worldwide, but more than 50 different genera of pathogens affect this species. Therefore, accelerating the improvement of fruit quality and pathogen resistance in strawberry represents an important objective for breeding and reducing the usage of pesticides. New genome sequencing data and bioinformatics tools has provided important resources to expand the use of synthetic biology-assisted intragenesis strategies as a powerful tool to accelerate genetic gains in strawberry. In this paper, we took advantage of these innovative approaches to create four RNAi intragenic silencing cassettes by combining specific strawberry new promoters and pathogen defense-related candidate DNA sequences to increase strawberry fruit quality and resistance by silencing their corresponding endogenous genes, mainly during fruit ripening stages, thus avoiding any unwanted effect on plant growth and development. Using a fruit transient assay, *GUS* expression was detected by the two synthetic *FvAAT2* and *FvDOF2* promoters, both by histochemical assay and qPCR analysis of GUS transcript levels, thus ensuring the ability of the same to drive the expression of the silencing cassettes in this strawberry tissue. The approaches described here represent valuable new tools for the rapid development of improved strawberry lines.

## 1. Introduction

Strawberry fruit (*Fragaria* spp.) is highly appreciated by consumers around the world and represents one of the most profitable crops of the *Rosaceae* family, whether in a fresh or processed form [1,2] (http://www.fao.org/faostat/en/#search/strawberries, accessed on 7 May 2021). In addition to sensorial attributes, such as colour, texture, aroma, and taste, which make this fruit very acceptable for human consumption, strawberries, like other berries, provide substantial benefits for health and blood sugar control, being an important source of manganese, potassium, folate (vitamin B9), vitamin C, and bioactive compounds, with high antioxidant capacity and potential cancer prevention effects [3,4,5,6]. However, characteristics such as firmness and vulnerability to pathogens significantly affect the yield and quality of the strawberry fruit, reducing its market value and consumption and are considered of great importance in breeding programs that seek to produce elite varieties with improved traits [7,8,9,10].

Among the most devastating pathogens in strawberry are fungi, with more than 50 different genera affecting this species [11,12]. Some of the major fungus-caused diseases include red stele disease (*Phytophthora fragariae*), Verticillium wilt (*Verticillium* spp.), gray mold (*Botrytis cinerea*), and anthracnose (*Colletotrichum* spp.). Under climate change, trends in the spread of crop pests and pathogens into new environments are increasing [13] and warmer temperatures have a dramatic effect on crop protection strategies, since it is affecting pathogen distribution and lifestyle and crop fitness and phenology [14]. At present, the global control of pathogens and pests of strawberry is mainly based on soil sterilization with fungicides, but their effectiveness for controlling diseases in fruiting fields is unclear [15]. Furthermore, plant protection products currently in use to protect strawberry and other crops are known to have potential undesirable side effects on human health and the environment and many of them will be phased out in the near future due to the increasing demand to reduce its application to crops [16,17,18,19,20]. Thus, it is of great interest to accelerate genetic resistance in this crop, since management of strawberry is expected to become more difficult under the influence of climate change and globalization.

Breeding for the improvement of strawberry is costly and time- and resource-intensive. Indeed, the genome of cultivated strawberry *Fragaria × ananassa* is octoploid, hampering traditional breeding, since many important traits, such as disease resistance, firmness, or taste, and aroma (among others), may be under the influence of multiple *loci* scattered over several subgenomes [21]. Over the past decade, a great effort has been made to unravel the genetic background of this species to help identify traits and associated genes of interest more quickly. The genome of *F. vesca* (diploid wild strawberry) has been completely sequenced and assembled, and subsequently, this genome information has been improved and reannotated [22,23,24,25,26,27]. Additionally, the genome of *F. × ananassa* (the octoploid cultivated species) cv. Camarosa has been completely sequenced and annotated, revealing its diploid progenitor species: *F. vesca* (subsp. Bracheata), *F. iinumae*, *F. nipponica*, and *F. viridis* [28]. This large contribution of strawberry genome data will greatly increase the efficiency of molecular marker-assisted breeding in this crop. However, strawberry genes controlling important traits remain unknown, and molecular marker technologies are limited [29]. Therefore, improvement of strawberry through traditional breeding is not expected to be so rapid.

Genetic modification strategies are faster at creating genetic variability than conventional breeding, adding “extra traits” that cannot be accessed by other traditional techniques [30,31]. In strawberry, targeted engineering of many traits, including some for resistance to diverse fungal pathogens, has been reported using these approaches. Successful enhanced resistance to *Sphaerotheca humuli*, *V. dahliae*, *P. cactorum*, *B. cinerea*, and *C. acutatum* has been described in transgenic strawberry, overexpressing genes from diverse origins, such as plant, fungal, or bacterial [32,33]. Genes include those encoding chitinase from rice [34,35], tomato (*Solanum chilense*) [36], and *Phaseolus vulgaris* [37], *thaumatin II* from *Thaumatococcus daniellii* [38], b-1,3-glucanase gene from *Trichoderma* [39], and *RolC* from *Agrobacterium rhizogenes* [40]. Even so, genetically modified organisms (GMOs) suffer from serious handicaps since they are not yet widely socially acceptable considering health and environmental concerns, due to scientifically unfounded misinformation and fearmongering campaigns [41]. However, using these molecular technologies over the past three decades, many crops have been successfully improved on beneficial agronomic and quality traits and many commercial GMOs have been rapidly adopted globally due to their contribution to food security, sustainability, reduction of agrochemical use, and climate change solutions, increasing ~112-fold from 1996, with an accumulated biotech area of 2.7 billion hectares [42,43,44,45]. Accordingly, at present, the introduction or modification of a single gene by directed gene transfer methods regains its value as a powerful tool to rapidly accelerate the improvement of superior varieties of strawberry and other woody fruit species.

In the past two decades, novelty and powerful biotechnology approaches have come to light to accurately, quickly, and efficiently address crop improvement, with fewer biosafety concerns. Therefore, new cisgenic and intragenic concepts have been introduced, these being much closer to traditional plant breeding methods, where only genes or DNA from the same or sexually compatible species can be incorporated in the plant [31,46,47,48]. Thus, in cisgenesis, genes containing their own native flanking regulatory regions, such a promoter and terminator in the normal-sense orientation, are added to the host organism [47]. Unlike cisgenic technology, intragenic technology allows the insertion and shuffling of different gene fragments. Thus, an intragenic plant can be originated by integrating into the plant genome a DNA cassette made of a combination of different gene fragments arranged in a sense or antisense orientation [47,49]. Intragenesis provides more recourses to modify gene expression and trait development than cisgenesis, since, by DNA shuffling, it is possible to create new genes (and therefore, proteins), including intragenes that target gene silencing (e.g., using RNAi cassettes). The European Food Safety Authority (EFSA) considers that hazards associated with cisgenic plants are similar to those associated with conventionally bred plants, whereas the putative unintended changes in intragenesis should be assessed on a case-by-case basis [50,51]. Additionally, modified crops through these approaches have much greater public acceptance than transgenesis, since they do not introduce foreign genes into the plant host genome, thus solving the current biosecurity problems related to this issue [52,53,54,55,56,57,58].

Although practical applications are still limited, following cisgenic and intragenic approaches, attempts to improve quality traits have been made in many crops, including barley, durum wheat, alfalfa, perennial ryegrass, poplar, potato, apple, grapevine, and strawberry [57,59,60,61,62,63,64,65,66,67,68,69,70,71,72,73,74,75,76,77,78,79]. Moreover, currently, cisgenic Arctic™ “Golden Delicious” and “Granny Smith” apples (Okanagan Specialty Fruits Inc., Summerland, BC, Canada), a cisgenic alfalfa with altered lignin production (Bayer, Germany), and the intragenic potatoes of the Innate™ line (J.R. Simplot Co., Boise, ID, USA) are cultivated for commercial purposes [53]. In strawberry, the only intragenic attempt so far was reported by Schaart and colleagues, combining the *FaPGIP* (a polygalacturonase inhibiting protein) and the promoter of the strawberry expansive gene, *FaEXP2*, which showed flower and fruit ripening specific expression [62]. However, intragenic strawberry plants did not show the expected enhanced resistance to this pathogen, highlighting the value of considering and evaluating new intragenic combinations with different strawberry promoters and candidate genes to achieve enhanced resistance of this crop to pathogens [62].

Accordingly, for an intragenic approach in strawberry, isolating and characterizing new promoters and valuable defense-related genes in this species acquires great relevance. So far, in addition to the *FaEXP2* promoter [62], few strawberry promoters have been isolated and characterized and include that of the root-specific *FaRB7* [80], those of the fruit-specific genes, *FanEG1* and *FanEG3*, which encode two endo-β-1,4-glucanases, respectively [81], *FaGalUR*, which encodes a D-galacturonate reductase [82], *FaSTAG1*, which encodes a MADS box protein [83], and those of the highly- and constitutively-expressed genes *FaGAPC1* (a glyceraldehyde 3-phosphate dehydrogenase), *FaUBCE2* (a ubiquitin conjugating enzyme), and *FaAPA1* (an aspartic proteinase precursor) [84]. However, with the progress in strawberry genome sequencing and the genome information already available for this species, identifying the gene sequences of interest and their regulatory motifs has become fairly easy, making intragenesis a very attractive and powerful tool to rapidly achieve strawberry improvement, while the undesirable effects associated with classical breeding process (‘linkage drag’) are also eliminated.

Additionally, genome sequence data has become an important resource to expand the use of synthetic biology (SynBio) to genetically modify strawberry. Indeed, synthetic DNA approaches have been applied with notable success in bacteria and yeast [85,86,87,88], and there are an increasing number of examples in plants, which include synthetic promoters, synthetic metabolic pathways, and synthetic genomes to modify and improve desirable traits in crops [89,90,91]. Currently, it is routine to synthesize DNA as long as 20–100 kb, providing the opportunity to easily engineer the assembly of DNA sequences of interest by modern gene synthesis methods [92,93]. Furthermore, since regulatory elements are interchangeable in intragenesis, a selective developmental or tissue expression of the intragenic construction of interest can be achieved by pre-design of the intragene “cassette” using promoters carrying regulatory elements of known and desired spatio-temporal expression pattern. This is particularly attractive for preventing side effects and unintended changes on the host plant. In addition, the de novo synthesis of DNA is particularly advantageous for an intragenic approach, since combination of native sequences, excluding foreign DNA sequences, can be limiting and difficult to achieve by traditional genetic engineering techniques.

In this paper, we report on the design and use of synthetic biology for the construction of four RNAi silencing cassettes by assembling specific DNA sequences of new strawberry ripening-related gene promoters and pathogen defense-related genes using an intragenic approach. Additionally, using promoter-GUS fusion reporter assays, the usefulness of these two chemically synthetized strawberry natural promoters and their upstream regulatory sequences has been evaluated in strawberry fruit via *Agrobacterium*-mediated transient experiments. Binary Ti-plasmids carrying the intragenic RNAi silencing cassettes have been constructed to conduct the silencing of the endogenous strawberry target defense-related genes precisely in fruit during the ripening process in order to avoid unwanted effects on plant growth and development. Moreover, the recipient binary plasmid frame carries the *Malus domestica MdMYB10* from a red-fleshed apple as a natural visual selectable marker [57,94], which will allow the production of stable red strawberry transformants (via *Agrobacterium* transformation), thus avoiding the integration into the strawberry genome of classical undesirable marker genes for selection. An increase in fruit resistance against different pathogens is expected in the strawberry lines carrying any of these RNAi silencing cassettes, which will benefit strawberry fruit yield and postharvest stages.

## 2. Results and Discussion

### 2.1. Creation of the Ti-Plasmid Constructs Carrying the Strawberry Intragenic dsRNA Silencing Cassette

**Figure 1:**
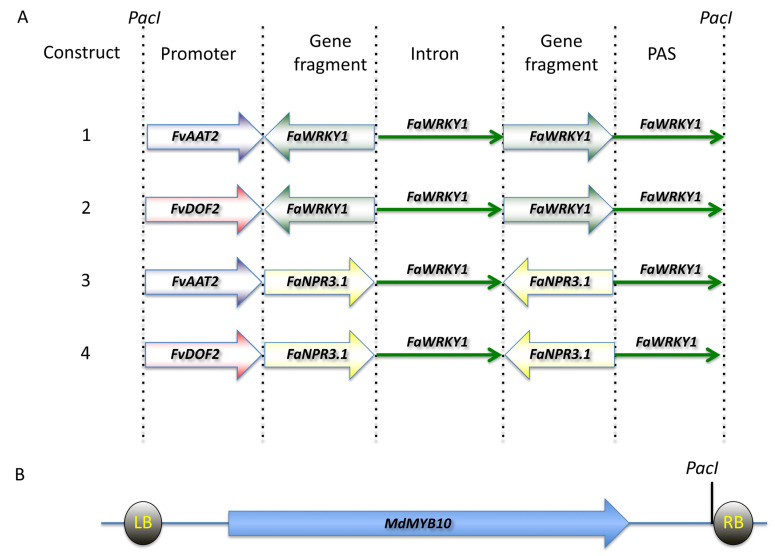
Creation of the Ti-plasmid constructs carrying the strawberry intragenic dsRNA silencing cassettes. (**A**) Design of the four intragenic-dsRNA-silencing cassettes. Promoter: promoter and regulatory sequences upstream from the ATG codon from genes *FvAAT2* and *FvDOF2*; Gene fragment: DNA fragment from genes *FaWRKY1* (272 bp) and *FaNPR3.1* (407 bp) used for their corresponding inverted target sequences; Intron: the unique intron sequence of *FaWRKY1* (652 bp); PAS: a 352 bp DNA fragment downstream from the stop codon of the *FaWRKY1*, containing the predicted regulatory signals for the cleavage and polyadenylation signal; *AscI* and *PacI*, restriction site sequences flanking the entire intragenic-dsRNA-silencing cassettes. (**B**) The T-region of plasmid pMinMYB carrying the complete genomic sequence of the apple *MdMYB10* gene (adapted from Krens et al. 2015). The position of the unique *PacI* restriction site used for cloning each of the four intragenic-dsRNA-silencing cassettes is shown. LB and RB, left and right border.

Four Ti-plasmid constructs were created using synthetic biology and an intragenic strategy, schematized in Figure 1. Each of them carries all the strawberry DNA sequences needed for a fruit ripening-related expression of an intragenic dsRNAi-inducing unit aimed to silence either of two relevant specific pathogen defense-related endogenous genes, *FaWRKY1* or *FaNPR3.1*.

#### 2.1.1. Identification and Selection of the Strawberry Candidate Defense-Related Target Genes FaWRKY1 and FaNPR3.1 and Design of the Intragenic dsRNAi-Inducing Unit

*FaWRKY1* was originally chosen as a target gene to silence, due to the fact that this strawberry gene has been described as an important element mediating defense responses to pathogens, and it is expressed in fruit after *C. acutatum* infection [95,96]. *FaWRKY1* is the strawberry ortholog of the *Arabidopsis AtWRKY75*, which encodes a type IIc member of the plant WRKY transcription factor family. Plant WRKY TFs are involved in controlling a wide range of physiological and developmental processes, including plant immunity, in which they play major roles [97,98,99]. In fact, *AtWRKY75* has been reported to show a positive regulatory role in defense when its overexpression enhanced *Arabidopsis* resistance to *Sclerotinia sclerotiorum* [100]. Interestingly, many WRKY TFs, including AtWRKY75-like factors, can exhibit a dual activity in plant defense according to the type of pathogen. For instance, the *AtWRKY75* ortholog in cotton, *GbWRKY1*, acts as a negative regulator of the JA-mediated defense response against the necrotrophic *B. cinerea* and the hemibiotrophic *V. dahliae* [101], whereas the *AtWRKY75* ortholog in grapevine, *VvWRKY1*, displays a positive regulatory function in defense against the biotrophic pathogen *Plasmopara viticola* [102]. Accordingly, in previous studies, it was demonstrated that *FaWRKY1* can act similarly to *AtWRKY75* as a positive regulator of defense in a heterologous system, such as *Arabidopsis,* either in compatible or incompatible interactions [95]. Very recently, it was also reported that the silencing of the *FaWRKY1* in strawberry fruit by *Agrobacterium*-mediated transient transformation enhanced fruit resistance to controlled *C. acutatum* infection compared to non-silenced fruit [103]. As for other *AtWRKY75*-like genes, a potential dual role was described for the strawberry *FaWRKY1*, which might evidence differences according to the pathogen lifestyle, but more importantly, these results demonstrated a relevant regulatory role of this strawberry gene in the mechanism of defense against *C. acutatum*, a major strawberry pathogen.

**Figure 2:**
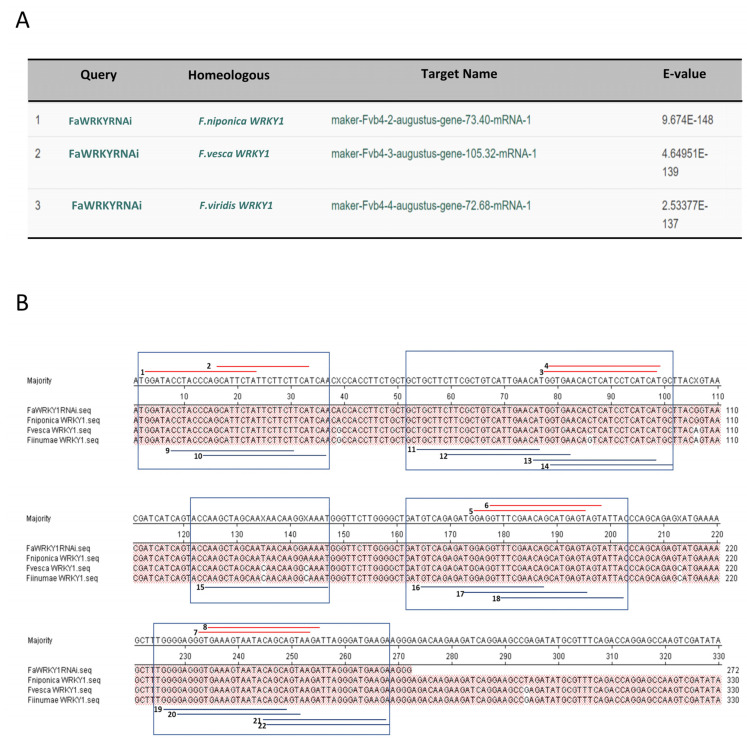
The FaWRKY1 DNA fragment selected for dsRNAi silencing (FaWRKYRNAi). (**A**) BLAST of the 272 bp DNA fragment of *FaWRKY1* against *Fragaria* × *ananassa* Camarosa Genome v1.0.a1 Transcripts database, adjusting the setting for the search to E-value 0.001 and Match/Mismatch Scores 1/−2. (**B**) Partial local alignment of the sequence, corresponding to three homeologs of *FaWRKY1* and the selected *FaWRKY1* DNA fragment. FaWRKY1RNAi corresponds to the 272 bp DNA fragment used in the intragenic dsRNAi-inducing unit; *F. niponica* WRKY1, maker-Fvb4-2-augustus-gene-73.40-mRNA-1; *F. vesca WRKY1*, maker-Fvb4-3-augustus-gene-105.32-mRNA-1; and *F. viridis* WRKY1, maker-Fvb4-4-augustus-gene-72.68-mRNA-1. Red and purple lines indicate the position of each predicted siRNA obtained by invivoGen software and Whitehead, respectively. Predicted siRNA are mostly grouped into “hot spot” RNAi candidate regions (boxes).

Therefore, a *FaWRKY1*-intragenic-dsRNAi-inducing unit was designed based on the following considerations: (a) the inverted target sequences correspond to the same 272 bp DNA fragment from the *FaWRKY1* described in Higuera-Sobrino et al. (2019), which was shown to successfully transiently silence the endogenous *FaWRKY1* in strawberry fruit, increasing the resistance of this tissue to *C. acutatum*; (b) the length of this *FaWRKY1* fragment is within the suitable size to maximize the efficiency of silencing [104] and its sequence does not drive cross-homology silencing, according to the criteria of Xu et al. (2006) (see next section below and Figure 2) [105]; (c) the sense and antisense *FaWRKY1* fragments were linked with a 652 bp DNA sequence fragment, corresponding to the native unique *FaWRKY1* intron sequence, aimed to act as an internal loop. The inclusion of this functional intron sequence in the sense orientation regarding the promoter is expected to have a consistently-enhancing silencing effect, as previously described [106,107,108].

The *FaNPR3.1* was also selected as a good defense-related candidate target gene to silence, as this strawberry gene seems to behave functionally similar to *At*NPR3/*At*NPR4 factors, with those who share a high degree of identity (Figure S1), which are members of the *Arabidopsis* non-expressor of pathogenesis-related (NPR) family of proteins involved in plant immunity, mediated by salicylic acid against biotrophic and hemi-biotrophic pathogens [109]. Indeed, *At*NPR3 and *At*NPR4 are the *Arabidopsis* paralogues of *At*NPR1, which acts as a positive master regulator of systemic acquired resistance, or SAR in this species [110,111], being also salicylic acid (SA) receptors but working redundantly as transcriptional corepressors of SA-mediated defense-related genes [112]. Recently, it has been reported that the strawberry *FvNPRL-1*, the *F. vesca FaNPR3.1* ortholog, displays a negative regulatory function of defense in *Arabidopsis* in response to biotic stresses [113]. Furthermore, preliminary analysis in strawberry fruit, with the endogenous *FaNPR3.1* transiently silenced via *Agrobacterium* transformation, has also revealed a significant decrease in the susceptibility of this tissue to the infection by *C. acutatum* [114].

**Figure 3:**
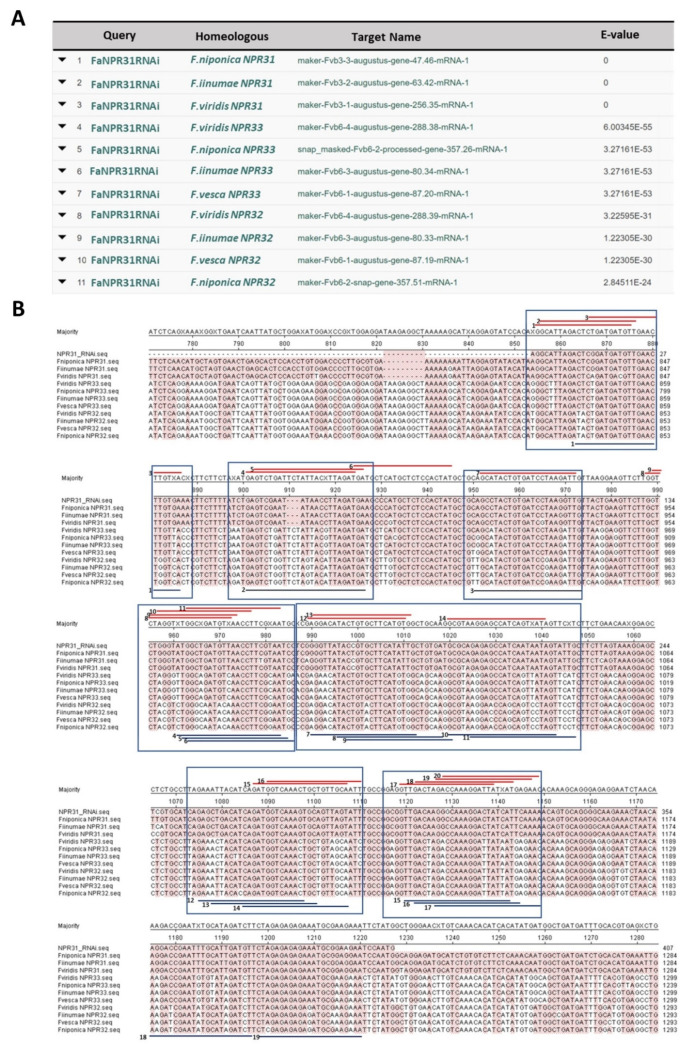
The FaNPR3.1 DNA fragment selected for dsRNAi silencing (FaNPR31RNAi). (**A**) BLAST of the 407 bp antisense DNA fragment of *FaNPR3.1* against *Fragaria × ananassa* Camarosa Genome v1.0.a1 Transcripts database, adjusting the setting for the search to E-value 0.001 and Match/Mismatch Scores 1/−2. (**B**) Partial local alignment of the sequence corresponding to three homeologs of *FaNPR3.1* and the selected DNA fragment. NPR31RNAi corresponds to the 407 bp DNA fragment used in the intragenic dsRNAi-inducing unit; *F. niponica* NPR31, maker-Fvb3-3-augustus-gene-47.46-mRNA-1; *F. iinumae* NPR31, maker-Fvb3-2-augustus-gene-63.42-mRNA-1; and *F. viridis* NPR31, maker-Fvb3-1-augustus-gene-256.35-mRNA-1. The corresponding homeologs to *FaNPR3.2* and *FaNPR3.3* are named as *F. viridis* NPR32, maker-Fvb6-4-augustus-gene-288.39-mRNA-1; *F. iinumae* NPR32, maker-Fvb6-3-augustus-gene-80.33-mRNA-1; *F. vesca* NPR32, maker-Fvb6-1-augustus-gene-87.19-mRNA-1; *F. niponica* NPR32, maker-Fvb6-2-snap-gene-357.51-mRNA-1; *F. viridis* NPR33, maker-Fvb6-4-augustus-gene-288.38-mRNA-1; *F. iinumae* NPR33, maker-Fvb6-3-augustus-gene-80.34-mRNA-1; *F. vesca* NPR33, maker-Fvb6-1-augustus-gene-87.20-mRNA-1; and *F. niponica* NPR33, snap_masked-Fvb6-2-processed-gene-357.26-mRNA-1. Red and purple lines indicate the predicted siRNA obtained by invivoGen software and Whitehead, respectively. Predicted siRNA are mostly grouped into “hot spot” RNAi candidate regions (boxes).

Thus, a *FaNPR3.1*-intragenic-dsRNAi-inducing unit was built on the following criteria: (a) the sense and antisense gene sequences were created using the same 407 bp DNA fragment from the *FaNPR3.1*, which successfully transiently silenced the endogenous target gene in strawberry fruit, increasing the resistance of this tissue to *C. acutatum* [114]; (b) since the selected 407 bp DNA sequence encompasses nearly identical *NPR3* gene alleles but no cross-homology with other members of the strawberry *FaNPR* gene family that has been detected, only silencing of all putative strawberry *FaNPR3* allele-specific transcripts is expected (see next section below and Figure 3); (c) similarly to FaWRKY1, the length of the *FaNPR3.1* DNA fragment is appropriate to maximize silencing efficiency [104] and enhance the silencing effect. The same original *FaWRKY1* splicable intron sequence of 652 bp already mention above, was interposed between the *FaNPR3.1*-inverted flanking target sequences [106,107,108].

#### 2.1.2. ‘‘Off-Target Effect” of the dsRNA Inducing Units

To understand unintended gene silencing of the intragenic dsRNA-inducing units on strawberry genes, the 272 bp and 407 bp DNA fragments of *FaWRKY1* and *FaNPR3.1*, respectively, were used as queries to perform nucleotide BLAST searches against the *Fragaria × ananassa* Camarosa Genome v1.0.a1 Transcripts database. Only the homoeologous genes scored E-values, with very high significance (Figure 2A, Figure 3A, Figures S2 and S3). To further explore any off-target effect, putative siRNAs were predicted within each DNA fragment by using specific software programs, which mimic the dicer activity on the induced dsRNA, providing a set of reliable 21–24 bp siRNAs (Figure 2B and Figure 3B). Thus, 8 and 14 candidate siRNA sequences were predicted, respectively, by the InvivoGen siRNA Wizard (https://www.invivogen.com/sirnawizard/design.php, accessed on 15 September 2021) and the siRNA at Whitehead (http://sirna.wi.mit.edu/home.php, accessed on 15 September 2021) free online software within the 272 bp *FaWRKY1* fragment (Figure S2A,B) and 20 and 19, respectively, within the selected 407 bp *FaNPR3.1* fragment (Figure S3A,B). Each of these predicted candidate siRNA sequences were used to BLAST against the *Fragaria × ananassa* Camarosa Genome v1.0.a1 Transcripts database, taking into account an upper limit of a contiguous nucleotide sequence of 18 bp to efficiently discriminate potential off-targets [105]. Only the *F. × ananassa* homoelogous genes were found with e-values of high significance, either for the *FaWRKY1* or the *FaNPR3.1* fragment (Figures S2C,D and S3C,D, respectively). Indeed, all the predicted siRNA candidates were easily grouped into a “hot spot” siRNA pattern within the *FaWRKY1* and *FaNPR3.1* homoeologous gene sequences (Figure 2B and Figure 3B), suggesting a great specificity of silencing of their corresponding intragenic dsRNA inducing units.

#### 2.1.3. Selection and Isolation of FvAAT2 and FvDOF2 Strawberry Promoter and Terminator Sequences

For specific dsRNAi production, the intragenic-dsRNAi-inducing-units were flanked with a promoter sequence from either of the two strawberry fruit ripening-related genes, *FvDOF2* and *FvAAT2* (*F. vesca* orthologs of *F. × ananassa FaAAT2* and *FaDOF2*, respectively), and a native *FaWRKY1* terminator region.

In *F. × ananassa*, the *FaAAT2* encodes a fruit-related acyltransferase involved in aroma biogenesis in fruit receptacles [115]. Indeed, the expression of *FaAAT2* is fruit-specific and increases during the strawberry fruit development and maturation stages, reaching high levels in red-mature and dark-red stages. Similarly, a higher expression was detected for the *FaDOF2* in strawberry fruit and petals of the octoploid cultivar compared to other tissues [116], according to the key role that *FaDOF2* transcription factor seems to play, together with FaEOBII, in the regulation of the phenylpropanoid volatile eugenol biosynthesis, which contributes to the aroma of fruit, as well as a floral attractant for pollinators. Moreover, the expression pattern of *FaDOF2* in strawberry fruit was receptacle-specific and ripening-dependent, increasing continuously from the green stage to over-red and senescence fruit stages [116]. Consequently, the regulatory regions of these strawberries *FaAAT2* and *FaDOF2* were considered valuable tools to control the expression of the intragenic dsRNAi-inducing units in fruit in a ripening-related manner, when this strawberry complex organ is more susceptible to pathogens, thus avoiding severe pleiotropic effects in plant growth and development. However, the regulatory regions of their corresponding orthologous genes in *F. vesca* were finally considered to control the expression of the intragenic-dsRNAi inducing unit. The reason for this is that the latest diploid *F. vesca* genome information available by the time of this intragenic design was extraordinarily improved over previous versions, and gene models and genome annotations were fully updated and were highly reliable [22,23,24,25,26,27], whereas a trustable near-complete chromosome-scale assembly of the cultivated strawberry (*F. × ananassa*) genome was not available yet; it was released later and only recently improved [28,117]. Additionally, it is known that *F. vesca* is the dominant subgenome, for cultivated strawberry and transcriptomic analyses have revealed that metabolic pathways giving rise to strawberry flavor, color, and aroma, are largely controlled by the dominant subgenome [28,118,119]. Accordingly, not only both *FaAAT2* and *FaDOF2 F. × ananassa* predicted homoeologous transcripts share high sequence identity between them and their corresponding *F. vesca* orthologs (see Figures S4 and S5), but a high sequence identity was also found between the regulatory regions of both *F. vesca* homoeologous *FaAAT2* and *FaDOF2* and the regulatory regions of their corresponding *F. vesca* ortholog genes (Figures S6 and S7, respectively). Most importantly, a similar fruit ripening-related gene expression pattern was previously detected for both *FaAAT2* and *FaDOF2* in *F. × ananassa* and their *FvAAT2* and *FvDOF2* orthologs in *F. vesca* [115] (Figure S8).

Consequently, the *F. vesca* genome was considered as the reference genome to select the regulatory sequence regions to control expression of the intragenic-dsRNAi inducing units. Therefore, DNA sequence fragments of 2998 bp and 3005 bp upstream of the predicted translation initiation codon (ATG) of genes *FvAAT2* (FvH4_5g24240) and *FvDOF2* (FvH4_2g14390), respectively, from *F. vesca* were selected as putative promoter sequences to drive the silencing cassettes in *Fx. ananassa* in a fruit ripening-related manner. The lengths of the selected regulatory regions are more in accordance with those described by Spolaore et al. (2003) than those described in Carvalho and Folta (2017), because larger strawberry promoter regions seem to display higher expression than shorter regions, which probably do not have the complete set of positive-acting elements and are under tight repression out of context [62,81,84,120]. We are aware that the expression pattern finally displayed by these *F. vesca* promoters in the octoploid strawberry fruit could be slightly modified, with respect to that expected, since it has already been reported that a large number of transcripts is altered between diploid and octoploid fruit during ripening [119].

Despite the importance that 3′ regulatory regions have for gene expression, they are still scarcely studied in plants compared to other regulatory sequences. The most widely used 3′ regulatory regions in plant expression vectors are *NOS* and *OCS* of *A. tumefaciens* and 35S of the cauliflower mosaic virus (CaMV); however, although still reduced, many plant 3′ regulatory sequences have already been described as good 3′ terminator regions, all having cis-elements involved in cleavage and polyadenylation [121]. So far, no 3′-end regulatory region has been validated for any strawberry gene, and only the terminator sequence of the ribulose biphosphate carboxylase small subunit gene (*MdRbcS*) of a crossable species, *Malus × domestica*, has been described [122]. However, for the intragenic approach considered here, the availability of suitable strawberry regulatory sequences is desirable. Therefore, the 352 bp DNA sequence downstream from the stop codon of the *FaWRKY1*, which carries all the necessary regulatory signals for RNA cleavage and polyadenylation, was considered as a good strawberry native 3′-UTR terminator region, and it was added to all constructs.

#### 2.1.4. Assembling the Intragenic dsRNA Silencing Cassettes

Each of the four intragenic-dsRNA_silencing-cassettes were assembled in silico according to the scheme shown in Figure 1A, and their entire sequences were chemically synthetized and cloned separately within the T-region of plasmid *pMinMYB* (Figure 1B). A complete genome sequence (including its promoter and terminator regions) of the apple *MdMYB10*, encoding a key transcription factor that regulates the expression of anthocyanin biosynthesis pathway genes, is also included within the RB-LB region of this plasmid. The *MdMYB10* has been reported as a useful red natural selectable marker in producing cisgenic apple [57,94].

### 2.2. Strawberry Promoter Analysis by Agrobacterium Mediated Transient Transformation

**Figure 4:**
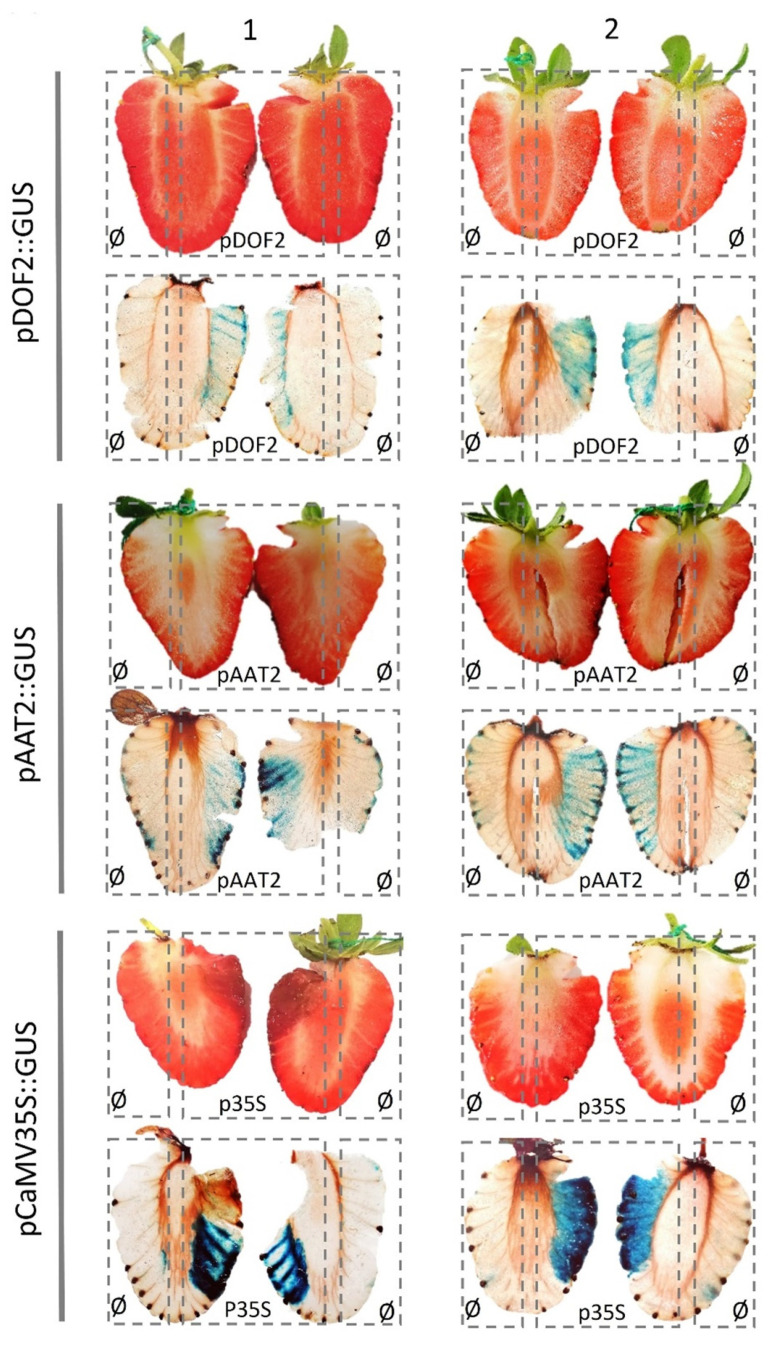
Transient promoter probe analysis of the synthetic *FvDOF2* and *FvAAT2* DNA fragments, respectively, in *F. × ananassa* fruit. Numbers **1** and **2** represent two different fruit samples. Histochemical GUS staining was performed after 5 days of fruit infiltration with agrobacterium carrying different query promoter constructs (*pFvDOF2*::GUS, *pFvAAT2*::GUS, and *pCaMV35s*::GUS) or the pKGWFS7.0 empty vector as a negative control (Ø). Query promoter constructs were injected in one half of the fruit, whereas the empty vector (Ø) was injected in the opposite half. For each construct, images represent tissues slices from two different fruit samples.

To confirm promoter activity in strawberry fruit of the candidate regulatory sequences obtained by synthetic biology from genes *FvAAT2* (FvH4_5g24240) and *FvDOF2* (FvH4_2g14390), both synthetic DNA fragments of 2998 bp and 3005 bp, respectively, upstream of the predicted translation initiation codon (ATG), were cloned into the promoter probe plasmid pKGWFS7.0 to drive the expression of the GUS reporter gene. Agrobacterium derivative strains harboring these constructs were then used in strawberry fruit transient experiment assays, using the agroinfiltration protocol described in Higuera et al. (2019) [103]. Histochemical assay of GUS activity revealed blue staining in fruit containing either *pFvAAT2*::GUS, *pFvDOF2*::GUS, or *pCaMV35S*::GUS (positive control) promoter constructs (Figure 4). No GUS staining was observed in strawberry fruit tissue infiltrated with the empty vector pKGWFS7.0. In all cases, blue staining was clearly visible and confined only within the half of fruit agroinfiltrated with the query promoter constructs but not within the opposite half agroinfiltrated with the empty vector. An uneven and patchy distribution of blue staining has been previously described by others and may be explained by the facility to diffuse by Agrobacterium according to the stage of fruit ripeness and the inherent variability in the assays [80,103]. Although the GUS staining technique is not accurate enough to report relative promoter strength, it was possible to distinguish a noticeable increase in blue intensity in the strawberry samples agroinfiltrated with the *pFvAAT2*::GUS construct, with respect to those with the *pFvDOF2*::GUS construct, the intensity being much higher in the *pCaMV35S*::GUS (positive control) samples.

**Figure 5:**
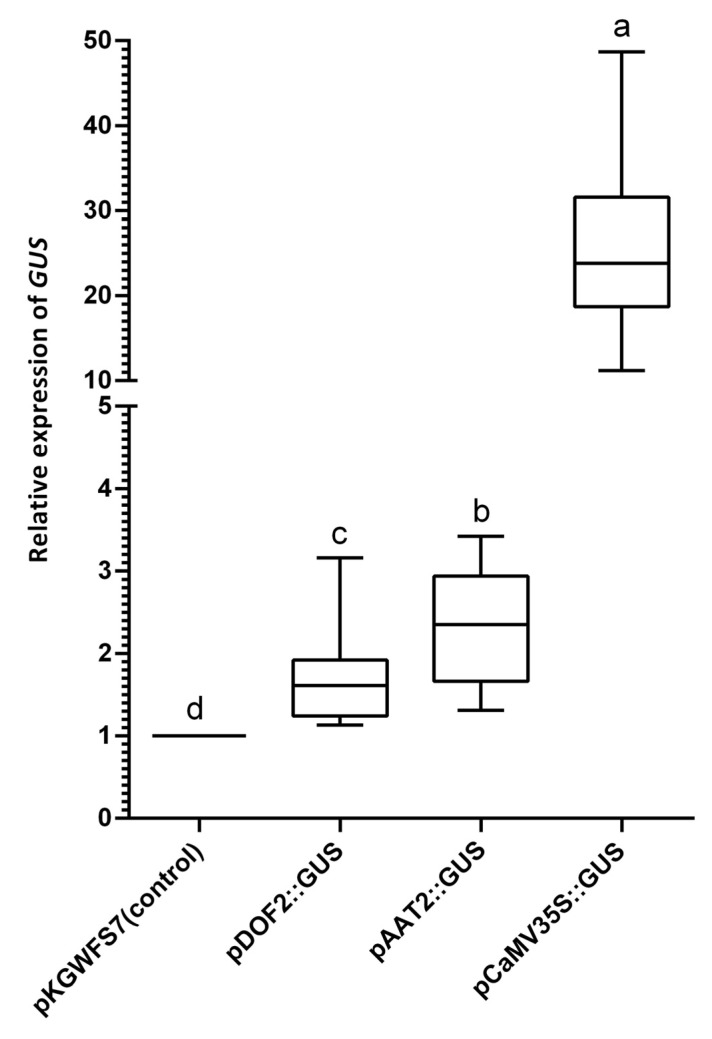
Quantitative real-time RT-qPCR expression analysis of GUS transcript levels in strawberry fruit transiently agroinfiltrated with the different promoter probe constructs. Box-plot graphs represent the distribution of gene expression values as relative GUS expression to the pKGWFS7 empty vector (negative control). Horizontal lines in the box indicate the median (Q2) and box the inter quartile range (Q3 Q1). Letters “a”, “b”, “c”, and “d” indicate statistically significant differences among samples, (*p* < 0.01, Wilcoxon–Mann–Whitney U-test).

Quantification of the corresponding GUS mRNA transcript levels by real-time RT-qPCR in strawberry fruit halves transiently expressing the corresponding *F. vesca* promoter constructs revealed that GUS transcripts are indeed significantly expressed in all the fruit samples agroinfiltrated with the query synthetic promoter constructs and confirmed that, although variable, under these conditions, the strength of the *pFvAAT2* promoter seems to be higher than the *pFvDOF2* promoter (Figure 5).

All in all, these results validate the use of these two synthetic promoters to drive the expression of the intragenic-dsRNAi-inducing units described in this paper in strawberry fruit. Additionally, these results demonstrate that *FvAAT2* and *FvDOF2* regulatory sequences from *F. vesca* are recognised in *F.* × *ananassa* fruit tissues and, most importantly, highlight synthetic biology as a powerful tool that can have a tremendous positive contribution to accelerate strawberry improvement.

## 3. Materials and Methods

### 3.1. Origin of DNA Sequence Fragments for the Intragenic dsRNAi Silencing Strategy

Strawberry intragenic silencing cassettes were designed based on a DNA blocks fusion scheme. Strawberry promoter sequences and their cis-acting regulatory signals were identified using the last version of the *F. vesca* Genome v4.0.a.1 of Rosacea Genome Database (https://www.rosaceae.org/, accessed on 12 October 2021). PlantCare (http://bioinformatics.psb.ugent.be/webtools/plantcare/html/, accessed on 12 October 2021) [123] and TSSplant v1.2016 (http://www.softberry.com, accessed on 12 October 2021) [124] were used to search for potential plant regulatory motifs, RNA Polymerase II TATA boxes, and transcription start sites. DNA fragments of 3005 bp and 2998 bp upstream of the predicted translation initiation codon (ATG) were retrieved for *FvDOF2* (FvH4_2g14390) and *FvAAT2* (FvH4_5g24240) genes, respectively (Figure S6). A 272 bp specific DNA fragment of *FaWRKY1* (maker-Fvb4-2-augustus-gene-73.40-mRNA-1), corresponding to the first 91 amino acids of FaWRKY1 protein, or a 407 bp specific DNA fragment of *FaNPR3.1* (maker-Fvb3-3-augustus-gene-47.46-mRNA-1), corresponding to 135 amino acids of the C-terminal region, was selected for the inverted regions of the “dsRNAi–inducing units”, respectively. In both cases, the sense and antisense DNA fragments were linked with the unique native 652 bp DNA intron sequence of the *FaWRKY1* as a spacer. Additionally, a genomic DNA sequence downstream of the predictable stop codon (TAG) of *FaWRKY1* was identified using the polyAH softberry program [125], and a 352 bp fragment was selected as a native 3′-UTR terminal region and added to both *FaWRKY1* and *FaNPR3.1* intragenic dsRNAi-producing units.

### 3.2. siRNA Predictions and “Off-Target” Effect

Candidate siRNA sequences were predicted using the InvivoGen_siRNA_Wizard (https://www.invivogen.com/sirnawizard/design.php, accessed on 12 October 2021) and the siRNA_at_Whitehead programs (http://sirna.wi.mit.edu/home.php, accessed on 12 October 2021). A contiguous segment of 18 bp was taken as a limit for off-target effect, following the criteria of Xu et al. (2006) [105].

### 3.3. Plasmid Constructs

The final entire assembled DNA sequence for each of the four strawberry intragenic silencing cassettes was chemically synthetized by GenScript Biotech company (Netherland) and cloned into the unique *PacI* site of the *pMinMYB* binary vector [57,126]. *pMinMYB* plasmid carries the complete genomic sequence of the *Malus domestica MdMYB10*, including its regulatory regions as a potential visible selectable marker in strawberry [94].

Two promoter probe vectors were constructed using the 3005 bp and 2989 bp DNA fragments, corresponding to the regulatory regions of *FvDOF2* and *FvAAT2* genes, respectively. Thus, these DNA fragments were PCR amplified using specific primers attB-FvDOF2full (attB1FvDOfII_Fw: GGGGACAAGTTTGTACAAAAAAGCAGGCTTGAACGTCATCGTAGCTTGC; attB2FvpDofII_Rv: GGGGACCACTTTGTACAAGAAAGCTGGGTTTTTGCAGAGAGGGTTTGGGT), for *FvDOF2* and attB-FvAAT2full (attB1FvAAT2_Fw GGGGACAAGTTTGTACAAAAAAGCAGGCTTTATTATGGAAAAGAATTGGTGAAGATGT; attB2-FvAAT2_Rv GGGGACCACTTTGTACAAGAAAGCTGGGTATCGATCACTAACACACAAGTACTCTC, for *FvAAT2*) from their corresponding synthetic DNA sequences obtained from Genscript and cloned independently into the Gateway^®^ entry vector *pDONR221* by gateway technology using standard protocols (Thermo Fischer scientific, (Waltham, MA, USA). Later, each of both promoter sequences were transferred to pKGWFS7.0 as a final destination vector carrying the *GUS* reporter gene [127]. pKGWFS7.0 was obtained from VIB Plant Systems Biology (Gent, Belgium).

*E. coli* XL10gold (Agilent Technologies Inc., Santa Clara, CA, USA) was used as a recipient for all these plasmids constructs, including their corresponding empty vectors. *A. tumefaciens* AGL0 [128] was used for strawberry transformation.

### 3.4. Promoter Probe Analysis and Strawberry Fruit Transient Experiments

The expression capacity of *FaAAT2* and *FaDOF2* synthetic promoters was analyzed in strawberry fruit by transient-agroinfiltration methodology and GUS reporter assays. The plasmid *pCaMV35s*::GUS previously described in Higuera et al. (2019) and the pKGWFS7.0 empty vector were used as positive and negative controls, respectively, in these experiments. Transient agroinfiltration was performed in strawberry fruit of the octoploid *F. × ananassa* (cv. M04502) at the pink/turning stage, following the protocol described in Higuera et al. (2019). Thus, turning strawberry fruits attached to the plant were agroinfiltrated in one half with 1 mL (slightly adjusted according to the size of the fruit) of a suspension of the *Agrobacterium* cells bearing either the query promoter construct or the *pCaMV35s*::GUS construct (positive control), whereas the opposite half of the same fruit received the *Agrobacterium*, bearing the corresponding empty vector, as a control. In this way, variability between fruit is reduced, so we are able to compare the results between halves of the same fruit in fruit with different ripening stages. All fruits were kept attached to the plant under natural growing conditions of light and temperature until harvesting. Fruit samples were collected 4–5 days after agroinfiltration, and samples from each of the two halves of the collected fruits were immediately used for histochemical GUS analysis or frozen in liquid nitrogen and transferred to −80 °C until use for the promoter expression analysis by RT-qPCR. A minimum of 6–9 fruits was sampled for each independent promoter analysis.

### 3.5. Histochemical Assay of GUS Activity

Strawberry transiently transformed fruit halves were used for the histochemical assay. GUS activity in strawberry fruit sections was performed, as described by Jefferson et al. (1987), using a modified staining solution, following the manufacturer (Gold Biotechnology, Saint Louis, MO, USA) instructions, containing: 2 mM X-gluc in 100 mM sodium phosphate buffer (pH 7.5), 10 mM EDTA, 0.1% (*v*/*v*) Triton X-100, and 1.0 mM potassium ferricyanide.

### 3.6. qRT-PCR Gene Expression Analyses and Statistical Analyses

Total RNA from frozen independent halves of the strawberry fruits agroinfiltrated was extracted with the Maxwell^®^ 16 LEV Plant RNA Kit (Promega, Madison, WI, USA), according to the instructions provided by the manufacturer. Total RNA was quantified by NanoDrop 1000 Spectrophotometer (Thermo Fischer scientific, Waltham, MA, USA), and RNA integrity (RIN) was checked using the Agilent 2100 Bioanalyzer (Agilent Technologies, Santa Clara, CA, USA). Reverse transcription (RT) was carried out using 250 ng of purified total RNA as a template from samples with a RIN value ≥ 8, for a 20 µL reaction [iScript cDNA Synthesis kit (Biorad, Hercules, CA, USA)]. RT-qPCR was performed using specific primers (Table S1) and SsoAdvancedTM SYBR^®^ Green Supermix in a CFX real-time PCR system (Bio-Rad, Hercules, CA, USA). All RT-qPCR primers used in this study had similar PCR efficiencies. The expression of the Kanamycin resistance gene was selected as an internal reference gene for GUS expression to normalize the level of transiently agroinfiltrated strawberry cells in every fruit. For each promoter probe analysis, six biological replicates, each with two RT technical replicates, were performed.

Fruit halves agroinfiltrated with *Agrobacterium* bearing the empty vector were used as control for statistical purposes. Thus, RT-qPCR GUS expression values were calculated and the mean was normalized as the relative expression value between the agroinfiltrated fruit half with the query promoter construct and the corresponding agroinfiltrated opposite fruit half with the empty control vector. Data were transformed into box-whisker plot graphics using the GraphPad Prism 8.0.2 program. The statistical value (*p*-value 0.001) was calculated using the Wilcoxon-Mann–Whitney U-test on data of two groups with a non-parametric statistical analysis. Additionally, a Student *t*-test was performed for pairwise comparisons between means of different groups. GUS values above 1 clearly indicate significant differences between both halves of the same fruit.

The expression analysis of the strawberry *FaDOF2* in green and red fruit receptacles of *F. vesca* and *F.* × *ananassa* cv. Camarosa was performed by qRT-PCR using specific primers for *FaDOF2* (Molina-Hidalgo et al., 2017).

## 4. Conclusions and Future Perspective

Intragenesis associated with the synthetic biology has emerged as a powerful approach to overcome the limitations and barriers of the traditional methods and speed up the improvement of strawberry. Using these strategies, four intragenic dsRNA silencing cassettes were designed, aimed to obtain stable strawberry lines with increased resistance/tolerance to different pathogens. Novel strawberry promoter sequences and candidate genes for silencing were considered. Synthetic *FvAAT2* and *FvDOF2* promoter sequences were validated as tools to drive the expression of *FaWRKY1* and *FaNPR3.1* dsRNAi-inducing units, mostly in strawberry fruit in a ripening related manner, featuring the relevance of synthetic biology to accelerate genetic improvement. Stable strawberry lines harboring each of these intragenic dsRNAi-silencing cassettes are expected to increase fruit resistance/tolerance to pathogens, and no serious interference in plant growth or any other tissues development stages is anticipated. We aimed to reduce the use of pesticides and unwanted compounds in agriculture. This approach could open the opportunity to increase fruit quality in strawberries by adding new traits in a faster way than conventional breeding methods.

## Data Availability

Data supporting this study are available within the paper and within its supplementary materials. Further information may be obtained from the corresponding authors.

## Supplementary Materials

The following are available online at https://www.mdpi.com/article/10.3390/plants11010057/s1, Figure S1: Phylogenetic tree and identity percentage of *NPR* gene family in strawberry and *A. thaliana*, Figure S2: Candidate DNA fragments of the strawberry *FaWRKY1* for dsRNA production (siRNAs), Figure S3: Candidate DNA fragments of the strawberry *FaNPR3.1* for dsRNA production (siRNAs), Figure S4: Alignment of the coding DNA sequence of the strawberry *AAT2* genes, Figure S5: Alignment of the coding DNA sequence of the strawberry *DOF2* genes, Figure S6: Alignment of promoter and regulatory region of the strawberry *AAT2* genes, Figure S7: Alignment of promoter and regulatory region of the strawberry *DOF2* genes, Figure S8. Expression analysis of the strawberry *FaDOF2* in green and red fruit receptacles of *F. vesca* and *F. × ananassa* cv. *Camarosa*. Table S1: Primers used in the RT-qPCR in this study.
