# Supplementary material for: The Intragenesis and Synthetic Biology Approach towards Accelerating Genetic Gains on Strawberry: Development of New Tools to Improve Fruit Quality and Resistance to Pathogens
Source: Plants (Basel). 2021 Dec 25;11(1):57. doi: 10.3390/plants11010057 (PMC8747664; doi:10.3390/plants11010057)
Supplement: Supplementary file 1 [file plants-11-00057-s001.zip › plants-1462547-supplementary.pdf]

plants

Article

The Intragenesis and Synthetic Biology Approach towards Accelerating Genetic Gains on Strawberry: Development of New Tools to Improve Fruit Quality and Resistance to Pathogens

Supplementary Materials:

Figure S1 Alignment of promoter and regulatory region of the strawberry AAT2 genes

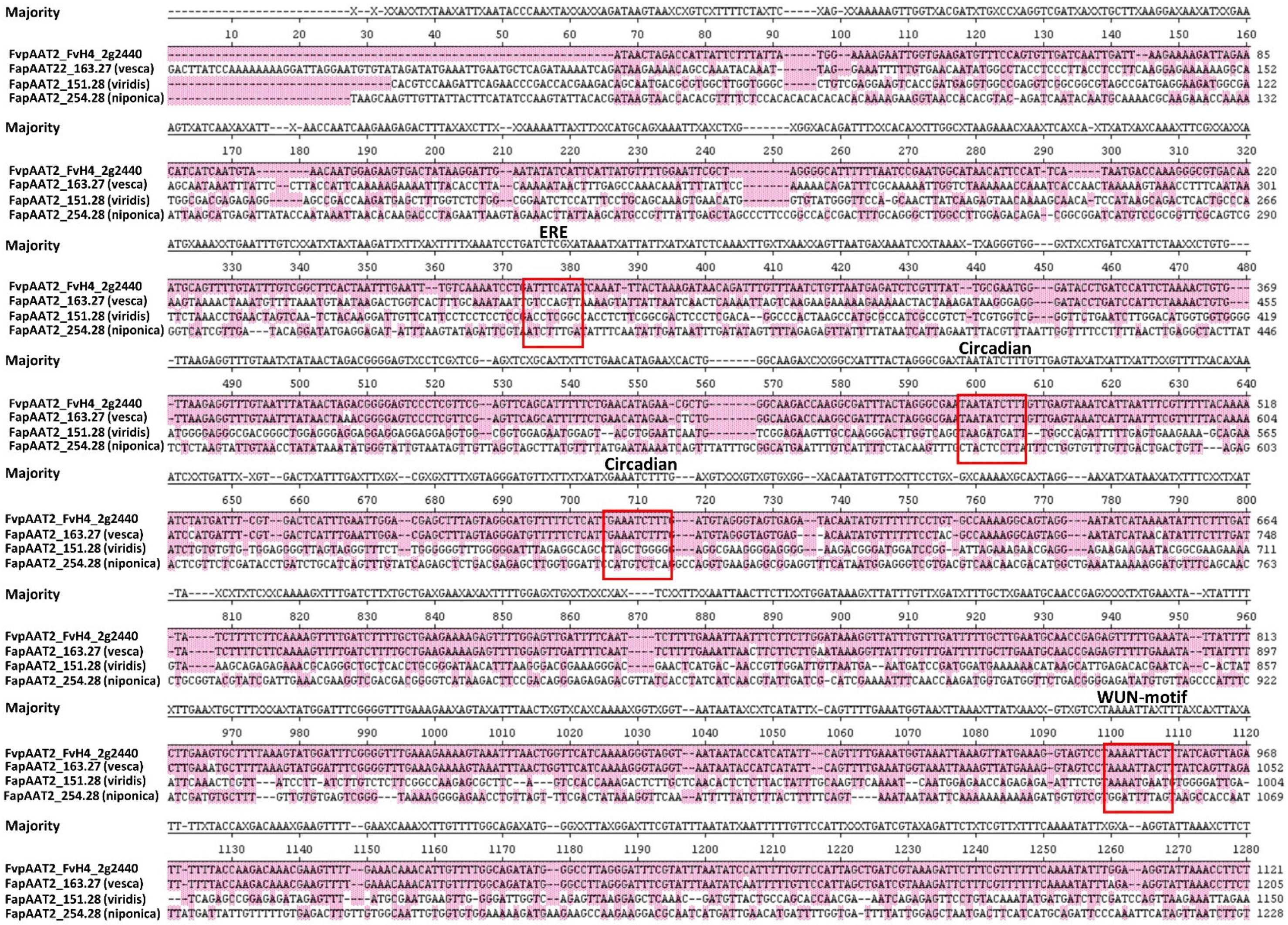

(Cont)

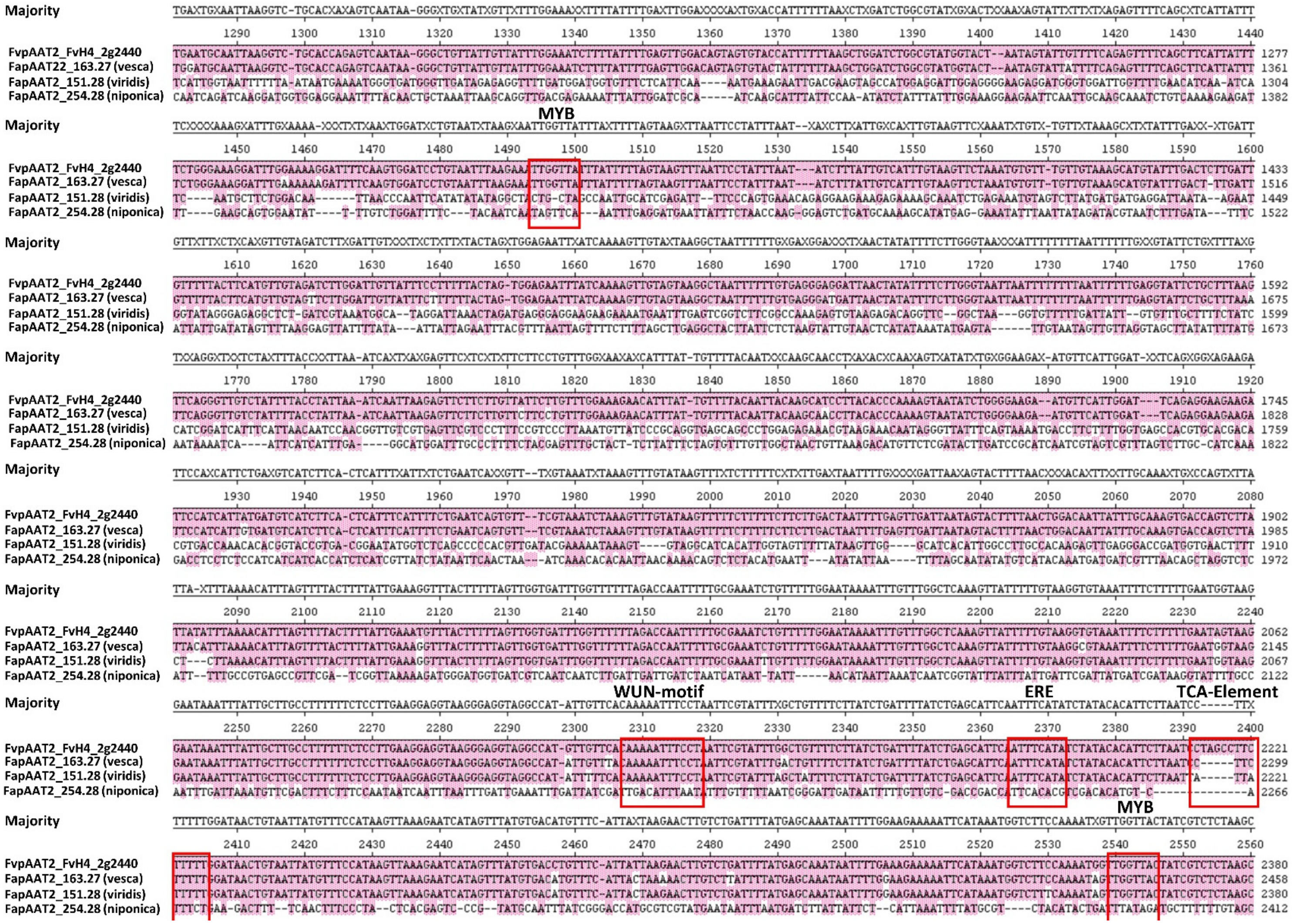
Majority
FvpAAT2_FvH4_2g2440
FapAAT22_163.27 (vesca)
FapAAT2_163.27 (vesca)
FapAAT2_151.28 (viridis)
FapAAT2_254.28 (niponica)
MYB
WUN-motif
ERE
TCA-Element

(Cont)

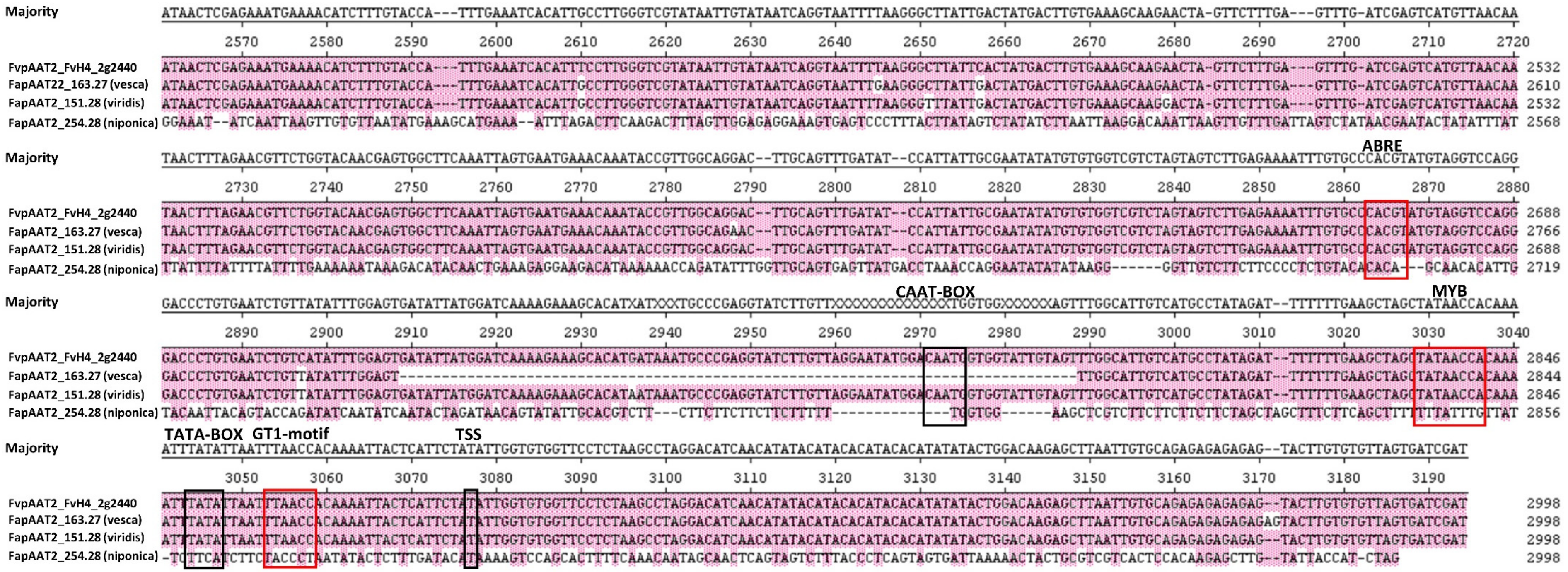

Figure S1: Alignment of promoter and regulatory region of the strawberry AAT2 genes*. Alignment of the 2998 bp DNA fragment corresponding to the promoter and regulatory region of FvAAT2 (FvH4_5g24240) with its respective homeologous of Fragaria x ananassa FapAAT2_163.27, maker-Fvb5-1-snap-gene-163.27-mRNA-1 (Vesca homeologous); FapAAT2_151.28, maker-Fvb5-2-snap-gene-151.28-mRNA-1 (Viridis homeologous); and FapAAT2_254.28 maker-Fvb3-3-augustus-gene-254.28-mRNA-1 (niponica homeologous). The putative RNA Polymerase II TATA box and transcription start sites (TSS) (****Black box****) and cis-acting regulatory motifs (****Red boxes****) were predicted by PlantCare (http://bioinformatics.psb.ugent.be/webtools/plantcare/html/) and TSSplant v1.2016 (http://www.softberry.com). All sequences were retrieved of Fragaria vesca v.4.0a1 and Fragaria x ananassa Camarosa Genome v1.0.a1 Transcripts database. ERE: Ethylene Response Element; ABRE: Abscisic acis Response element; WUN-motif: wounding-responsive element; TCA element: SA-responsive elements.*

Figure S2 Alignment of promoter and regulatory region of the strawberry DOF2 genes

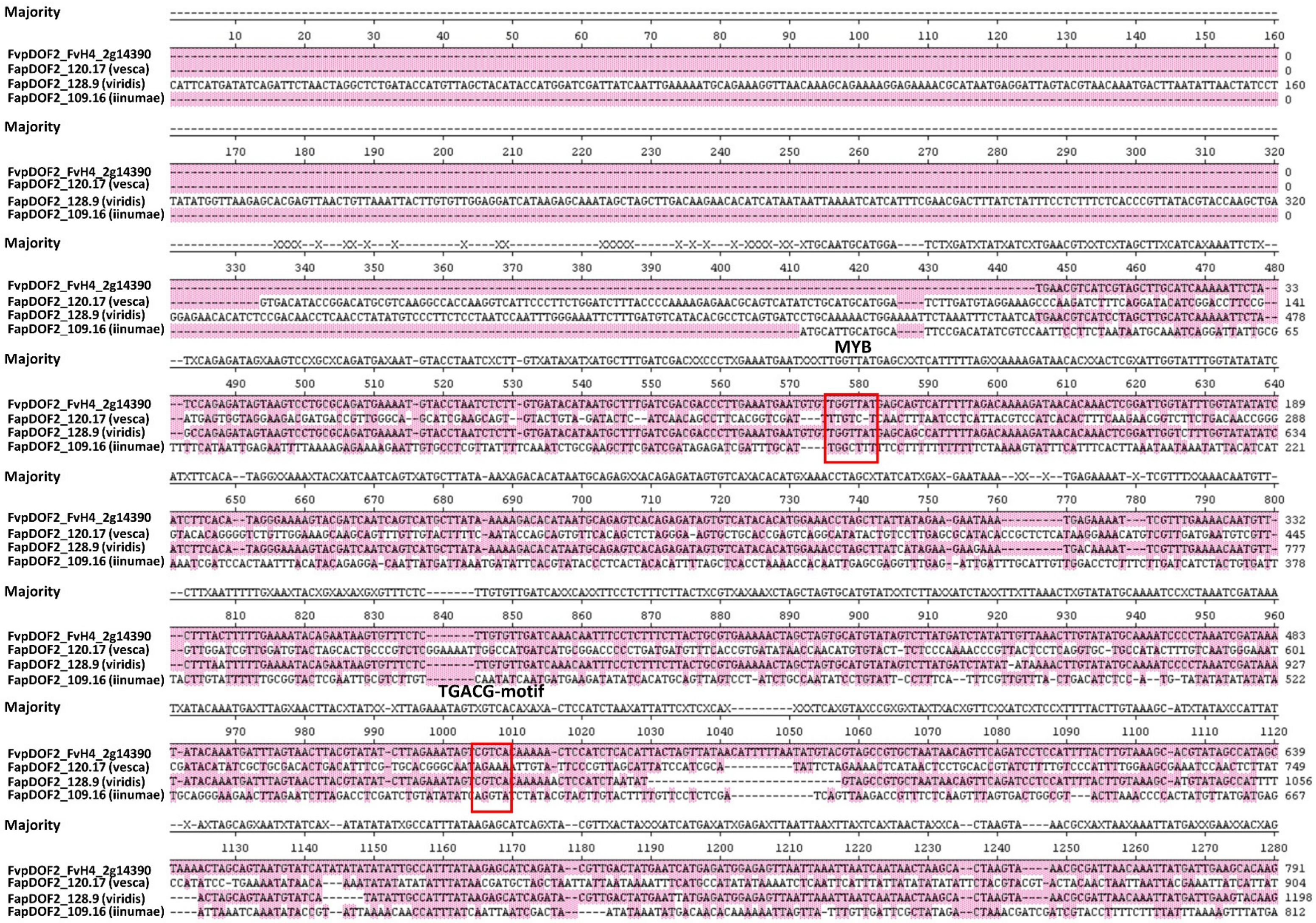

(Cont)

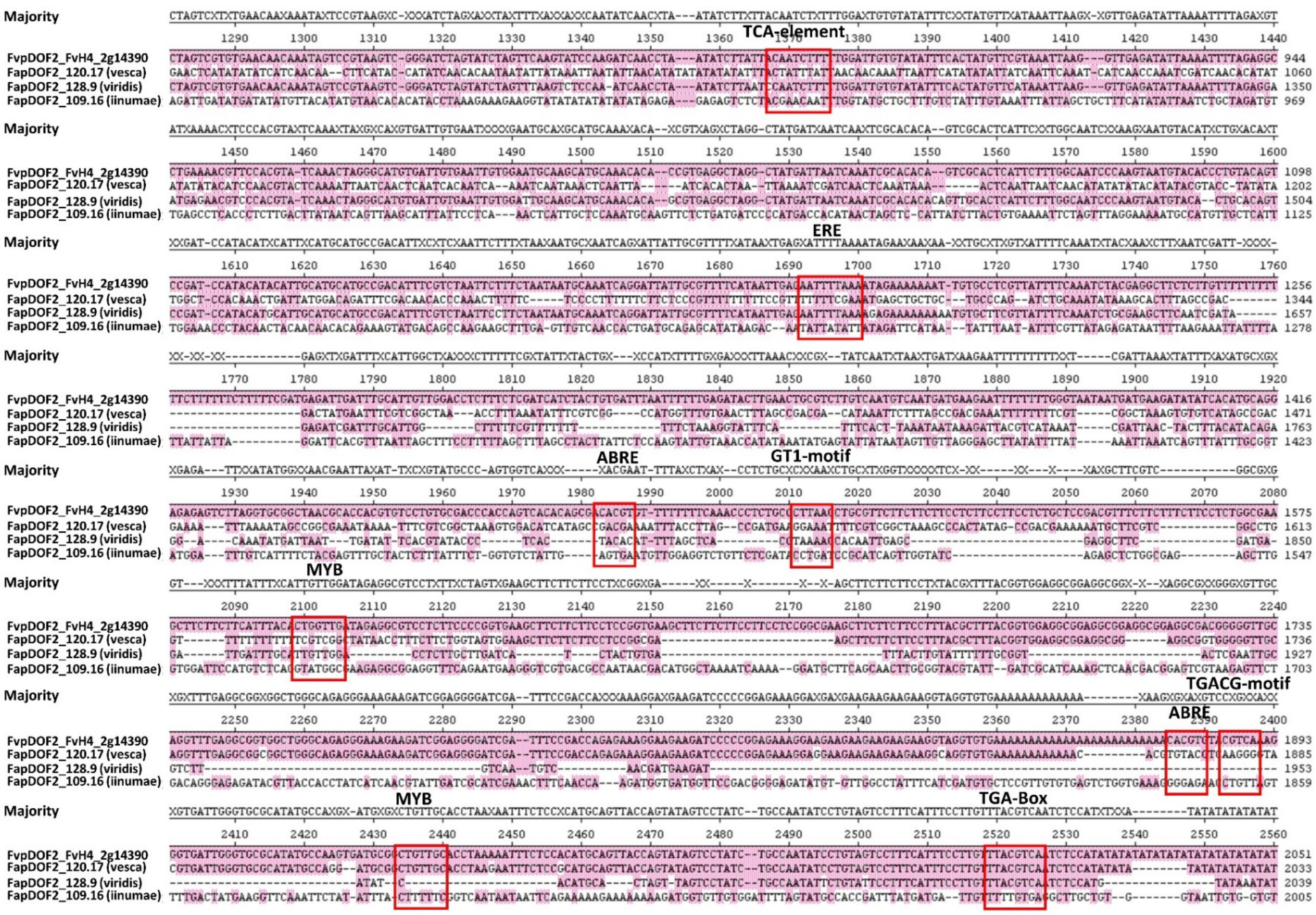
Majority
FvpDOF2_FvH4_2g14390
FapDOF2_120.17 (vesca)
FapDOF2_128.9 (viridis)
FapDOF2_109.16 (iinumae)
TCA-element
ERE
ABRE
GT1-motif
MYB
TGACG-motif
TGA-Box

(Cont)

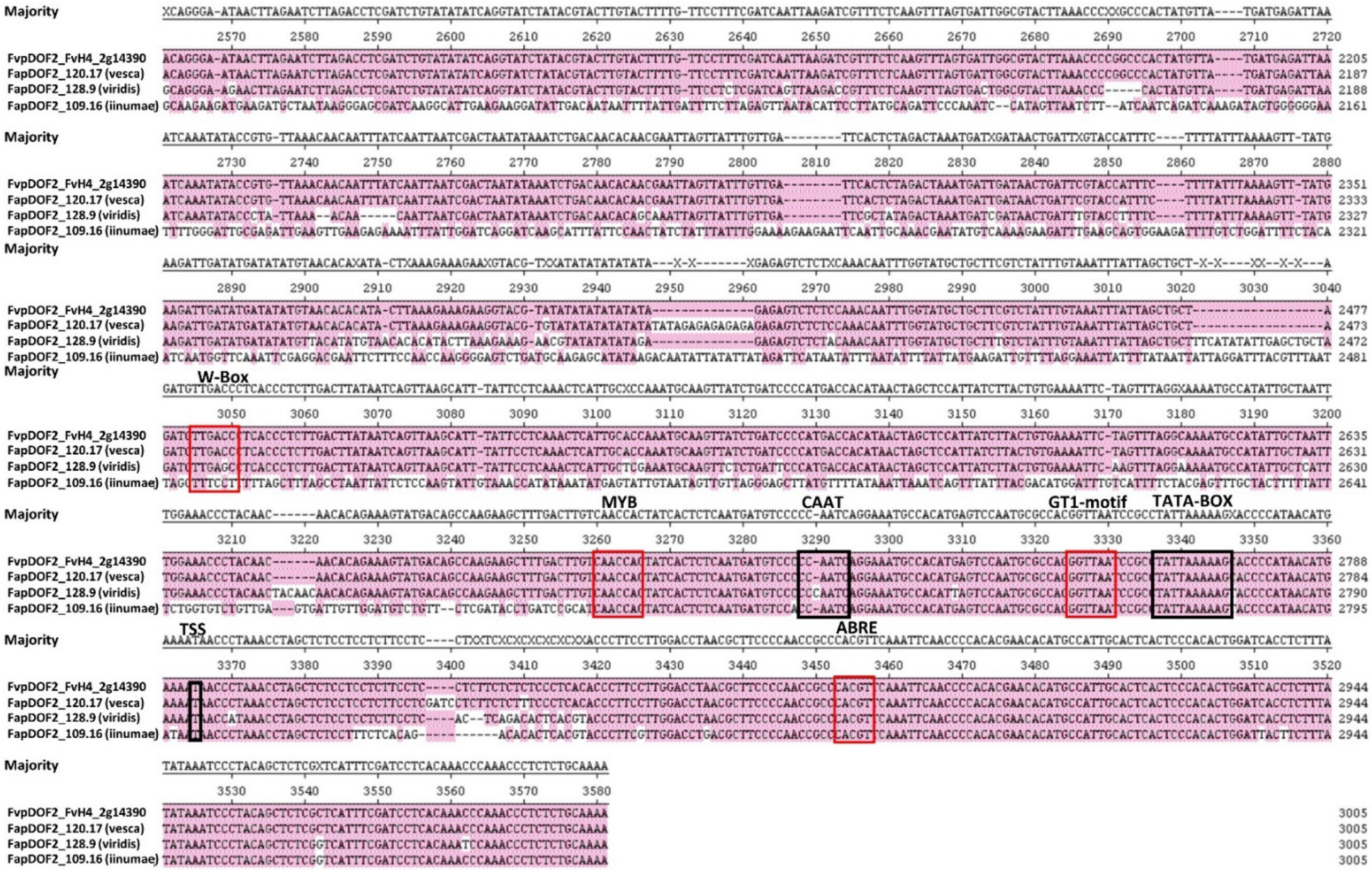

***Figure S2: Alignment of promoter and regulatory region of the strawberry DOF2 genes**. Alignment of the 3005 bp DNA fragment corresponding to promoter and regulatory region of the FvDOF2 (FvH4_2g14390) with its respective homeologous of Fragaria x ananassa FapDOF2_120.17, snap_masked-Fvb2-2-processed-gene-120.17-mRNA-1 (Vesca homeologous); FapDOF2_128.9, augustus_masked-Fvb2-3-processed-gene-128.9-mRNA-1(Viridis homeologous); and FapDOF2_109.16 snap_masked-Fvb2-4-processed-gene-109.16-mRNA-1 (iinumae homeologous). The putative RNA Polymerase II TATA box and transcription start sites (TSS) (**Black box**) and cis-acting regulatory motif (**Red boxes**) were predicted by*

PlantCare (http://bioinformatics.psb.ugent.be/webtools/plantcare/html/) and TSSplant v1.2016 (http://www.softberry.com). All sequences were retrieved of Fragaria vesca v.4.0a1 and Fragaria x ananassa Camarosa Genome v1.0.a1 Transcripts database. ERE: Ethylene Response Element; ABRE: Abscisic acis Response element; WUN-motif: wounding-responsive element; TCA element: SA-responsive elements.

Figure S3 Phylogenetic tree and identity percentage of NPR gene family in strawberry and A. thaliana

A

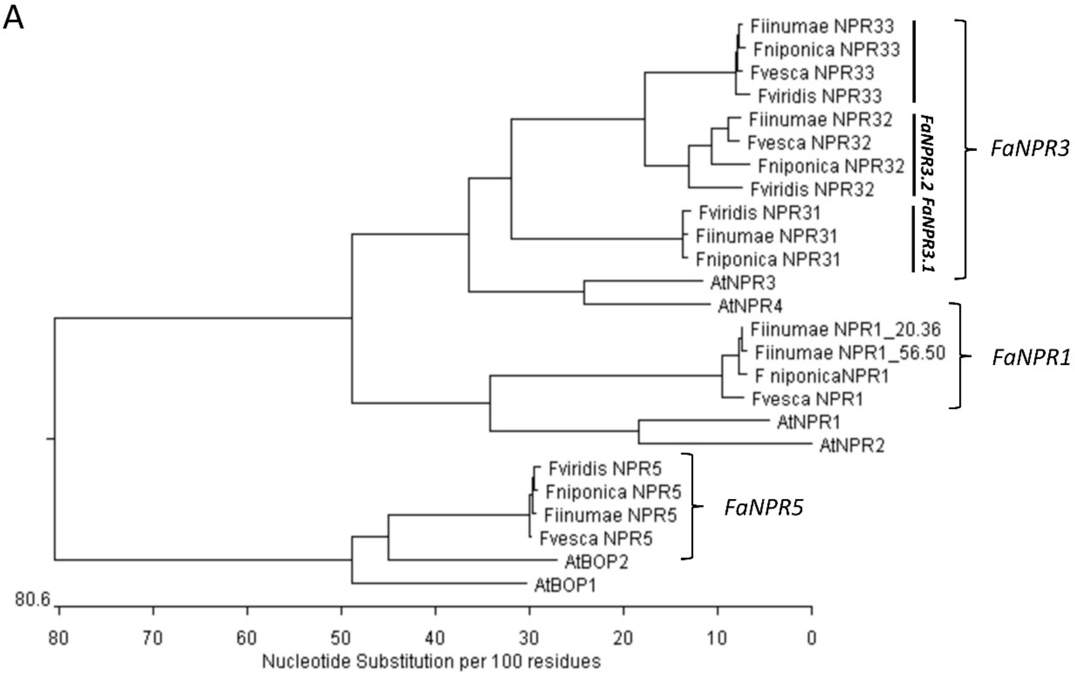

B

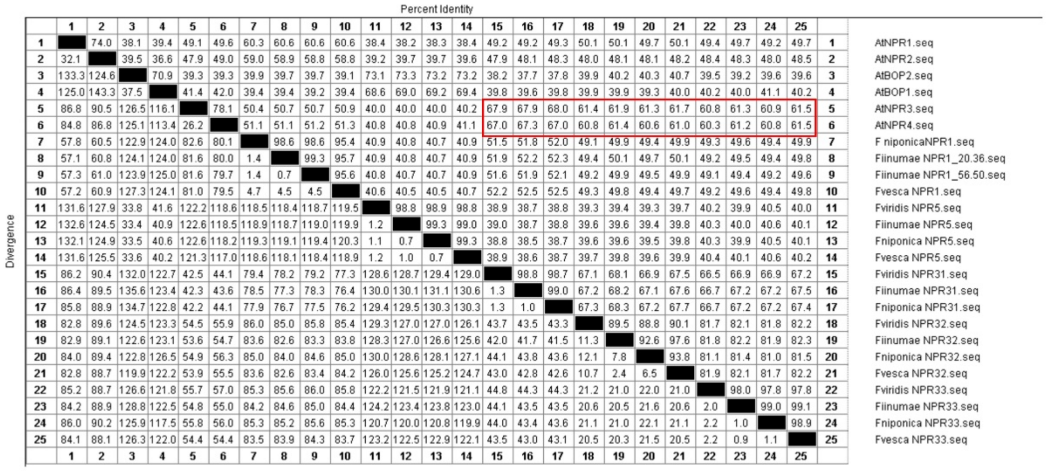

Percent Identity

Divergence

	1	2	3	4	5	6	7	8	9	10	11	12	13	14	15	16	17	18	19	20	21	22	23	24	25		
1		74.0	38.1	39.4	49.1	49.6	60.3	60.6	60.6	60.6	38.4	38.2	38.3	38.4	49.2	49.2	49.3	50.1	50.1	49.7	50.1	49.4	49.7	49.2	49.7	1	AtNPR1.seq
2	32.1		39.5	36.6	47.9	49.0	59.0	58.9	58.8	58.8	39.2	39.7	39.7	39.6	47.9	48.1	48.3	48.0	48.1	48.1	48.2	48.4	48.3	48.0	48.5	2	AtNPR2.seq
3	133.3	124.6		70.9	39.3	39.3	39.9	39.7	39.7	39.1	73.1	73.3	73.2	73.2	38.2	37.7	37.8	39.9	40.2	40.3	40.7	39.5	39.2	39.6	39.6	3	AtBOP2.seq
4	125.0	143.3	37.5		41.4	42.0	39.4	39.4	39.2	39.4	68.6	69.0	69.2	69.4	39.8	39.6	39.8	39.9	39.9	39.3	40.0	40.2	40.0	41.1	40.2	4	AtBOP1.seq
5	86.8	90.5	126.5	116.1		78.1	50.4	50.7	50.7	50.9	40.0	40.0	40.0	40.2	67.9	67.9	68.0	61.4	61.9	61.3	61.7	60.8	61.3	60.9	61.5	5	AtNPR3.seq
6	84.8	86.8	125.1	113.4	26.2		51.1	51.1	51.2	51.3	40.8	40.8	40.9	41.1	67.0	67.3	67.0	60.8	61.4	60.6	61.0	60.3	61.2	60.8	61.5	6	AtNPR4.seq
7	57.8	60.5	122.9	124.0	82.6	80.1		98.6	98.6	95.4	40.9	40.8	40.7	40.9	51.5	51.8	52.0	49.1	49.9	49.4	49.9	49.3	49.6	49.4	49.9	7	F niponicaNPR1.seq
8	57.1	60.8	124.1	124.0	81.6	80.0	1.4		99.3	95.7	40.9	40.8	40.7	40.9	51.9	52.2	52.3	49.4	50.1	49.7	50.1	49.2	49.5	49.4	49.8	8	Fiinumae NPR1_20.36.seq
9	57.3	61.0	123.9	125.0	81.6	79.7	1.4	0.7		95.6	40.8	40.7	40.7	40.9	51.6	51.9	52.1	49.2	49.9	49.5	49.9	49.1	49.4	49.2	49.6	9	Fiinumae NPR1_56.50.seq
10	57.2	60.9	127.3	124.1	81.0	79.5	4.7	4.5	4.5		40.6	40.5	40.5	40.7	52.2	52.5	52.5	49.3	49.8	49.4	49.7	49.2	49.6	49.4	49.8	10	Fvesca NPR1.seq
11	131.6	127.9	33.8	41.6	122.2	118.6	118.5	118.4	118.7	119.5		98.8	98.9	98.8	38.9	38.7	38.8	39.3	39.4	39.3	39.7	40.2	39.9	40.5	40.0	11	Fviridis NPR5.seq
12	132.6	124.5	33.4	40.9	122.6	118.5	118.9	118.7	119.0	119.9	1.2		99.3	99.0	39.0	38.7	38.8	39.6	39.6	39.4	39.8	40.3	40.0	40.6	40.1	12	Fiinumae NPR5.seq
13	132.1	124.9	33.5	40.6	122.6	118.2	119.3	119.1	119.4	120.3	1.1	0.7		99.3	38.8	38.5	38.7	39.6	39.6	39.5	39.8	40.3	39.9	40.5	40.1	13	Fniponica NPR5.seq
14	131.6	125.5	33.6	40.2	121.3	117.0	118.6	118.1	118.4	118.9	1.2	1.0	0.7		38.9	38.6	38.7	39.7	39.8	39.6	39.9	40.4	40.1	40.6	40.2	14	Fvesca NPR5.seq
15	86.2	90.4	132.0	122.7	42.5	44.1	79.4	78.2	79.2	77.3	128.6	128.7	129.4	129.0		98.8	98.7	67.1	68.1	66.9	67.5	66.5	66.9	66.9	67.2	15	Fviridis NPR31.seq
16	86.4	89.5	135.6	123.4	42.3	43.6	78.5	77.3	78.3	76.4	130.0	130.1	131.1	130.6	1.3		99.0	67.2	68.2	67.1	67.6	66.7	67.2	67.2	67.5	16	Fiinumae NPR31.seq
17	85.8	88.9	134.7	122.8	42.2	44.1	77.9	76.7	77.5	76.2	129.4	129.5	130.3	130.3	1.3	1.0		67.3	68.3	67.2	67.7	66.7	67.2	67.2	67.4	17	Fniponica NPR31.seq
18	82.8	89.6	124.5	123.3	54.5	55.9	86.0	85.0	85.8	85.4	129.3	127.0	127.0	126.1	43.7	43.5	43.3		89.5	88.8	90.1	81.7	82.1	81.8	82.2	18	Fviridis NPR32.seq
19	82.9	89.1	122.6	123.1	53.6	54.7	83.6	82.6	83.3	83.8	128.3	127.0	126.6	125.6	42.0	41.7	41.5	11.3		92.6	97.6	81.8	82.2	81.9	82.3	19	Fiinumae NPR32.seq
20	84.0	89.4	122.8	126.5	54.9	56.3	85.0	84.0	84.6	85.0	130.0	128.6	128.1	127.1	44.1	43.8	43.6	12.1	7.8		93.8	81.1	81.4	81.0	81.5	20	Fniponica NPR32.seq
21	82.8	88.7	119.9	122.2	53.9	55.5	83.6	82.6	83.4	84.2	126.0	125.6	125.2	124.7	43.0	42.8	42.6	10.7	2.4	6.5		81.9	82.1	81.7	82.2	21	Fvesca NPR32.seq
22	85.2	88.7	126.6	121.8	55.7	57.0	85.3	85.6	86.0	85.8	122.2	121.5	121.9	121.1	44.8	44.3	44.3	21.2	21.0	22.0	21.0		98.0	97.8	97.8	22	Fviridis NPR33.seq
23	84.2	88.9	128.8	122.5	54.8	55.0	84.2	84.6	85.0	84.4	124.2	123.4	123.8	123.0	44.1	43.5	43.5	20.6	20.5	21.6	20.6	2.0		99.0	99.1	23	Fiinumae NPR33.seq
24	86.0	90.2	125.9	117.5	55.8	56.0	85.3	85.2	85.6	85.3	120.7	120.0	120.8	119.9	44.0	43.4	43.6	21.1	21.0	22.1	21.1	2.2	1.0		98.9	24	Fniponica NPR33.seq
25	84.1	88.1	126.3	122.0	54.4	54.4	83.5	83.9	84.3	83.7	123.2	122.5	122.9	122.1	43.5	43.0	43.1	20.5	20.3	21.5	20.5	2.2	0.9	1.1		25	Fvesca NPR33.seq
	1	2	3	4	5	6	7	8	9	10	11	12	13	14	15	16	17	18	19	20	21	22	23	24	25		

Figure S3. Phylogenetic tree and identity percentage of NPR gene family in strawberry and A. thaliana*. *(A) *Phylogenetic analysis of five members of Fragaria ananassa NPR gene family and six member of A. thaliana. The sequences were aligned and analyzed by clustalW method with 1000 bootstrap replications. Numbers on branches are the bootstrap values. The scale bar at the bottom indicates the evolutionary distance.* ***(B)*** *Divergence and percentage identity of nucleotide sequences of NPR gene family between F.x ananassa and Arabidopsis thaliana. The nucleotide sequence of AtNPR3/4 present the similar percentage (around 60 to 70%) of homologous with respect to Fragaria NPR3 gene family* **(Red Box).** *Fragaria ananassa sequences correspond to Fiinumae NPR1_20.36, maker-Fvb1-2-augustus-gene-20.36-mRNA-1; Fiinumae NPR1_56.50, maker-Fvb1-2-augustus-gene-56.50-mRNA-1; Fvesca NPR1, maker-Fvb1-4-augustus-gene-33.69-mRNA-1; Fniponica NPR1, maker-Fvb1-3-augustus-gene-37.59-mRNA-1;Fniponica NPR31, maker-Fvb3-3-augustus-gene-47.46-*

mRNA-1; Fiinumae NPR31, maker-Fvb3-2-augustus-gene-63.42-mRNA-1 and Fviridis NPR31, maker-Fvb3-1-augustus-gene-256.35-mRNA-1. The corresponding homeolog to FaNPR32 and FaNPR33 are named as Fviridis NPR32, maker-Fvb6-4-augustus-gene-288.39-mRNA-1; Fiinumae NPR32, maker-Fvb6-3-augustus-gene-80.33-mRNA-1; Fvesca NPR32, maker-Fvb6-1-augustus-gene-87.19-mRNA-1; Fniponica NPR32, maker-Fvb6-2-snap-gene-357.51-mRNA-1; Fviridis NPR33, maker-Fvb6-4-augustus-gene-288.38-mRNA-1; Fiinumae NPR33, maker-Fvb6-3-augustus-gene-80.34-mRNA-1; Fvesca NPR33, maker-Fvb6-1-augustus-gene-87.20-mRNA-1 and Fniponica NPR33, snap_masked-Fvb6-2-processed-gene-357.26-mRNA-1; Fvesca NPR5, maker-Fvb6-1-augustus-gene-182.42-mRNA-1; Fniponica NPR5, maker-Fvb6-2-augustus-gene-104.36-mRNA-1; Fviridis NPR5, maker-Fvb6-3-augustus-gene-192.35-mRNA-1; Fiinumae NPR5, maker-Fvb6-4-augustus-gene-184.25-mRNA-1. For the Arabidosis NPR sequence, NPR1: At1g64280; NPR2: At4g26120; NPR3At5g45110; NPR4: At4g19660;NPR5(BOP2);At2g41370; NPR6(BOP1);At3g572130.

FigureS4 Candidate DNA fragments of the strawberry FaWRKY1 gene for dsRNA production (siRNAs).

A

№	Sequence (5´-3´)	Start
1	GGATACCTACCCAGCATTCTA	3
2	GCATTCTATTCTTCTTCATCA	16
3	GGTGAACACTCATCCTCATCA	78
4	GTGAACACTCATCCTCATCAT	79
5	GAGGTTTCGAACAGCATGAGT	175
6	GTTTCGAACAGCATGAGTAGT	178
7	GTGAAAGTAATACAGCAGTAA	213
8	GAAAGTAATACAGCAGTAAGA	215

C

№	siRNA	Homeologous	Target Name	E-value	ID
1	1	*F.viridisWRKY1*	maker-Fvb4-4-augustus-gene-72.68-mRNA-1	0.000819148	21
2	1	*F.vescaWRKY1*	maker-Fvb4-3-augustus-gene-105.32-mRNA-1	0.000819148	21
3	1	*F.niponicaWRKY1*	maker-Fvb4-2-augustus-gene-73.40-mRNA-1	0.000819148	21
4	2	*F.viridisWRKY1*	maker-Fvb4-4-augustus-gene-72.68-mRNA-1	0.000819148	21
5	2	*F.vescaWRKY1*	maker-Fvb4-3-augustus-gene-105.32-mRNA-1	0.000819148	21
6	2	*F.niponicaWRKY1*	maker-Fvb4-2-augustus-gene-73.40-mRNA-1	0.000819148	21
7	3	*F.vescaWRKY1*	maker-Fvb4-3-augustus-gene-105.32-mRNA-1	0.000819148	21
8	3	*F.niponicaWRKY1*	maker-Fvb4-2-augustus-gene-73.40-mRNA-1	0.000819148	21
9	4	*F.vescaWRKY1*	maker-Fvb4-3-augustus-gene-105.32-mRNA-1	0.000819148	21
10	4	*F.niponicaWRKY1*	maker-Fvb4-2-augustus-gene-73.40-mRNA-1	0.000819148	21
11	5	*F.viridisWRKY1*	maker-Fvb4-4-augustus-gene-72.68-mRNA-1	0.000819148	21
12	5	*F.vescaWRKY1*	maker-Fvb4-3-augustus-gene-105.32-mRNA-1	0.000819148	21
13	5	*F.niponicaWRKY1*	maker-Fvb4-2-augustus-gene-73.40-mRNA-1	0.000819148	21
14	6	*F.viridisWRKY1*	maker-Fvb4-4-augustus-gene-72.68-mRNA-1	0.000819148	21
15	6	*F.vescaWRKY1*	maker-Fvb4-3-augustus-gene-105.32-mRNA-1	0.000819148	21
16	6	*F.niponicaWRKY1*	maker-Fvb4-2-augustus-gene-73.40-mRNA-1	0.000819148	21
17	7	*F.viridisWRKY1*	maker-Fvb4-4-augustus-gene-72.68-mRNA-1	0.000819148	21
18	7	*F.vescaWRKY1*	maker-Fvb4-3-augustus-gene-105.32-mRNA-1	0.000819148	21
19	7	*F.niponicaWRKY1*	maker-Fvb4-2-augustus-gene-73.40-mRNA-1	0.000819148	21
20	8	*F.viridisWRKY1*	maker-Fvb4-4-augustus-gene-72.68-mRNA-1	0.000819148	21
21	8	*F.vescaWRKY1*	maker-Fvb4-3-augustus-gene-105.32-mRNA-1	0.000819148	21
22	8	*F.niponicaWRKY1*	maker-Fvb4-2-augustus-gene-73.40-mRNA-1	0.000819148	21

B

№	Sequence (5´-3)	Start
9	ACCTACCCAGCATTCTATTCTT	7
10	CAGCATTCTATTCTTCTTCATCA	14
11	TGCTTCTTCGCTGTCATTGAACA	54
12	TTCGCTGTCATTGAACATGGTGA	60
13	ATGGTGAACACTCATCCTCATCA	76
14	GTGAACACTCATCCTCATCATGC	79
15	AAGCTAGCAATAACAAGGAAAAT	125
16	GTCAGAGATGGAGGTTTCGAACA	165
17	TGGAGGTTTCGAACAGCATGAGT	173
18	TTCGAACAGCATGAGTAGTATTA	180
19	GGGGAGGGTGAAAGTAATACAGC	226
20	GAGGGTGAAAGTAATACAGCAGT	229
21	CAGCAGTAAGATTAGGGATGAAG	245
22	CAGCAGTAAGATTAGGGATGAAG	246

D

№	siRNA	*Homeologous*	Target Name	E-value	ID
1	9	*F.viridisWRKY1*	maker-Fvb4-4-augustus-gene-72.68-mRNA-1	6.71529E-5	23
2	9	*F.vescaWRKY1*	maker-Fvb4-3-augustus-gene-105.32-mRNA-1	6.71529E-5	23
3	9	*F.niponicaWRKY1*	maker-Fvb4-2-augustus-gene-73.40-mRNA-1	6.71529E-5	23
4	10	*F.viridisWRKY1*	maker-Fvb4-4-augustus-gene-72.68-mRNA-1	6.71529E-5	23
5	10	*F.vescaWRKY1*	maker-Fvb4-3-augustus-gene-105.32-mRNA-1	6.71529E-5	23
6	10	*F.niponicaWRKY1*	maker-Fvb4-2-augustus-gene-73.40-mRNA-1	6.71529E-5	23
7	11	*F.viridisWRKY1*	maker-Fvb4-4-augustus-gene-72.68-mRNA-1	6.71529E-5	23
8	11	*F.vescaWRKY1*	maker-Fvb4-3-augustus-gene-105.32-mRNA-1	6.71529E-5	23
9	11	*F.niponicaWRKY1*	maker-Fvb4-2-augustus-gene-73.40-mRNA-1	6.71529E-5	23
10	12	*F.viridisWRKY1*	maker-Fvb4-4-augustus-gene-72.68-mRNA-1	6.71529E-5	23
11	12	*F.vescaWRKY1*	maker-Fvb4-3-augustus-gene-105.32-mRNA-1	6.71529E-5	23
12	12	*F.niponicaWRKY1*	maker-Fvb4-2-augustus-gene-73.40-mRNA-1	6.71529E-5	23
13	13	*F.vescaWRKY1*	maker-Fvb4-3-augustus-gene-105.32-mRNA-1	6.71529E-5	23
14	13	*F.niponicaWRKY1*	maker-Fvb4-2-augustus-gene-73.40-mRNA-1	6.71529E-5	23
15	14	*F.vescaWRKY1*	maker-Fvb4-3-augustus-gene-105.32-mRNA-1	6.71529E-5	23
16	14	*F.niponicaWRKY1*	maker-Fvb4-2-augustus-gene-73.40-mRNA-1	6.71529E-5	23
17	15	*F.vescaWRKY1*	maker-Fvb4-2-augustus-gene-73.40-mRNA-1	6.71529E-5	23
18	16	*F.viridisWRKY1*	maker-Fvb4-4-augustus-gene-72.68-mRNA-1	6.71529E-5	23
19	16	*F.vescaWRKY1*	maker-Fvb4-3-augustus-gene-105.32-mRNA-1	6.71529E-5	23
20	16	*F.niponicaWRKY1*	maker-Fvb4-2-augustus-gene-73.40-mRNA-1	6.71529E-5	23
21	17	*F.viridisWRKY1*	maker-Fvb4-4-augustus-gene-72.68-mRNA-1	6.71529E-5	23
22	17	*F.vescaWRKY1*	maker-Fvb4-3-augustus-gene-105.32-mRNA-1	6.71529E-5	23
21	17	*F.niponicaWRKY1*	maker-Fvb4-2-augustus-gene-73.40-mRNA-1	6.71529E-5	23
24	18	*F.viridisWRKY1*	maker-Fvb4-4-augustus-gene-72.68-mRNA-1	6.71529E-5	23
25	18	*F.vescaWRKY1*	maker-Fvb4-3-augustus-gene-105.32-mRNA-1	6.71529E-5	23
26	18	*F.niponicaWRKY1*	maker-Fvb4-2-augustus-gene-73.40-mRNA-1	6.71529E-5	23
27	19	*F.viridisWRKY1*	maker-Fvb4-4-augustus-gene-72.68-mRNA-1	6.71529E-5	23
28	19	*F.vescaWRKY1*	maker-Fvb4-3-augustus-gene-105.32-mRNA-1	6.71529E-5	23
29	19	*F.niponicaWRKY1*	maker-Fvb4-2-augustus-gene-73.40-mRNA-1	6.71529E-5	23
30	20	*F.viridisWRKY1*	maker-Fvb4-4-augustus-gene-72.68-mRNA-1	6.71529E-5	23
31	20	*F.vescaWRKY1*	maker-Fvb4-3-augustus-gene-105.32-mRNA-1	6.71529E-5	23
32	20	*F.niponicaWRKY1*	maker-Fvb4-2-augustus-gene-73.40-mRNA-1	6.71529E-5	23
33	21	*F.viridisWRKY1*	maker-Fvb4-4-augustus-gene-72.68-mRNA-1	6.71529E-5	23
34	21	*F.vescaWRKY1*	maker-Fvb4-3-augustus-gene-105.32-mRNA-1	6.71529E-5	23
35	21	*F.niponicaWRKY1*	maker-Fvb4-2-augustus-gene-73.40-mRNA-1	6.71529E-5	23
36	22	*F.viridisWRKY1*	maker-Fvb4-4-augustus-gene-72.68-mRNA-1	6.71529E-5	23
37	22	*F.vescaWRKY1*	maker-Fvb4-3-augustus-gene-105.32-mRNA-1	6.71529E-5	23
38	22	*F.niponicaWRKY1*	maker-Fvb4-2-augustus-gene-73.40-mRNA-1	6.71529E-5	23

Figure S4: ***Candidate DNA fragments of the strawberry FaWRKY1 for dsRNA production (siRNAs)***. *(**A**) and (**B**) indicate the sequence and the position of each predicted siRNA within the 272 bp DNA fragment of the FaWRKY1 used for the construction of the corresponding intragenic dsRNAi inducing cassettes, and (**C**) and (**D**) BLAST of each candidate dsRNAi inducing sequence against v1.0.a1 Transcripts database indicating the identity with its corresponding homoeologous in the Fragaria ananassa genome. Predicted siRNA sequence were obtained by using the InvivoGen siRNA Wizard software 3.1.(A) and siRNA at Whitehead (B). Search parameters of the BLAST search against Fragaria x ananassa Camarosa Genome transcripts database were adjusted to the short sequence (e-value 0,05 and Match/Mismatch Scores 2/-3).*

FigureS5 Candidate DNA fragments of the strawberry FaNPR31 gene for dsRNA production (siRNAs).

A

№	Sequence (5´-3´)	Start
1	GGCATTAGACTCGGATGATGT	2
2	GCATTAGACTCGGATGATGTT	3
3	GGATGATGTTGAACTTGTGAA	14
4	GAGTCGAATATAACCTTAGAT	48
5	GTCGAATATAACCTTAGATGA	50
6	GAAGCCCATGCTCTCCACTAT	69
7	GCCTACTGTGATCCTAAGGTT	96
8	GGTCTGGGTATGGCTGATGTT	132
9	GTCTGGGTATGGCTGATGTTA	133
10	GGGTATGGCTGATGTTAACCT	137
11	GCTGATGTTAACCTTCGTAAT	144
12	GGGTTATACCGTGCTTCATAT	170
13	GGTTATACCGTGCTTCATATT	171
14	GCGCAGAGAGCCATCAATAAT	200
15	GATGGTCAAAGTGCAGTTAGT	267
16	GGTCAAAGTGCAGTTAGTATT	270
17	GTTGACAAGGGCAAAGGACTA	299
18	GACAAGGGCAAAGGACTATCA	302
19	GGGCAAAGGACTATCATTCAA	307
20	GGCAAAGGACTATCATTCAAA	308

C

№	siRNA	Homeologous	Target Name	E-value	ID
1	1	*F.iinumaeNPR31*	maker-Fvb3-2-augustus-gene-63.42-mRNA-1	0.000819148	21
2	1	*F.niponicaNPR31*	maker-Fvb3-3-augustus-gene-47.46-mRNA-1	0.000819148	21
3	2	*F.iinumaeNPR31*	maker-Fvb3-2-augustus-gene-63.42-mRNA-1	0.000819148	21
4	2	*F.niponicaNPR31*	maker-Fvb3-3-augustus-gene-47.46-mRNA-1	0.000819148	21
5	3	*F.iinumaeNPR31*	maker-Fvb3-2-augustus-gene-63.42-mRNA-1	0.000819148	21
6	3	*F.niponicaNPR31*	maker-Fvb3-3-augustus-gene-47.46-mRNA-1	0.000819148	21
7	4	*F.iinumaeNPR31*	maker-Fvb3-2-augustus-gene-63.42-mRNA-1	0.000819148	21
8	4	*F.niponicaNPR31*	maker-Fvb3-3-augustus-gene-47.46-mRNA-1	0.000819148	21
9	4	*F.viridisNPR31*	maker-Fvb3-1-augustus-gene-256.35-mRNA-1	0.000819148	21
10	5	*F.iinumaeNPR31*	maker-Fvb3-2-augustus-gene-63.42-mRNA-1	0.000819148	21
11	5	*F.niponicaNPR31*	maker-Fvb3-3-augustus-gene-47.46-mRNA-1	0.000819148	21
12	5	*F.viridisNPR31*	maker-Fvb3-1-augustus-gene-256.35-mRNA-1	0.000819148	21
13	6	*F.iinumaeNPR31*	maker-Fvb3-2-augustus-gene-63.42-mRNA-1	0.000819148	21
14	6	*F.niponicaNPR31*	maker-Fvb3-3-augustus-gene-47.46-mRNA-1	0.000819148	21
15	7	*F.iinumaeNPR31*	maker-Fvb3-2-augustus-gene-63.42-mRNA-1	0.000819148	21
16	7	*F.niponicaNPR31*	maker-Fvb3-3-augustus-gene-47.46-mRNA-1	0.000819148	21
17	8	*F.iinumaeNPR31*	maker-Fvb3-2-augustus-gene-63.42-mRNA-1	0.00997927	18
18	8	*F.niponicaNPR31*	maker-Fvb3-3-augustus-gene-47.46-mRNA-1	0.00997927	18
19	8	*F.viridisNPR31*	maker-Fvb3-1-augustus-gene-256.35-mRNA-1	0.00997927	18
20	9	*F.iinumaeNPR31*	maker-Fvb3-2-augustus-gene-63.42-mRNA-1	0.00285911	19
21	9	*F.niponicaNPR31*	maker-Fvb3-3-augustus-gene-47.46-mRNA-1	0.00285911	19
22	9	*F.viridisNPR31*	maker-Fvb3-1-augustus-gene-256.35-mRNA-1	0.00285911	19
23	10	*F.iinumaeNPR31*	maker-Fvb3-2-augustus-gene-63.42-mRNA-1	0.000819148	21
24	10	*F.niponicaNPR31*	maker-Fvb3-3-augustus-gene-47.46-mRNA-1	0.000819148	21
25	10	*F.viridisNPR31*	maker-Fvb3-1-augustus-gene-256.35-mRNA-1	0.000819148	21
26	11	*F.niponicaNPR31*	maker-Fvb3-3-augustus-gene-47.46-mRNA-1	0.000819148	21
27	11	*F.viridisNPR31*	maker-Fvb3-1-augustus-gene-256.35-mRNA-1	0.000819148	21
28	12	*F.iinumaeNPR31*	maker-Fvb3-2-augustus-gene-63.42-mRNA-1	0.000819148	21
29	12	*F.viridisNPR31*	maker-Fvb3-1-augustus-gene-256.35-mRNA-1	0.000819148	21
30	13	*F.iinumaeNPR31*	maker-Fvb3-2-augustus-gene-63.42-mRNA-1	0.000819148	21
31	13	*F.viridisNPR31*	maker-Fvb3-1-augustus-gene-256.35-mRNA-1	0.000819148	21
32	14	*F.iinumaeNPR31*	maker-Fvb3-2-augustus-gene-63.42-mRNA-1	0.000819148	21
33	14	*F.niponicaNPR31*	maker-Fvb3-3-augustus-gene-47.46-mRNA-1	0.000819148	21
34	15	*F.iinumaeNPR31*	maker-Fvb3-2-augustus-gene-63.42-mRNA-1	0.000819148	21
35	15	*F.niponicaNPR31*	maker-Fvb3-3-augustus-gene-47.46-mRNA-1	0.000819148	21
36	15	*F.viridisNPR31*	maker-Fvb3-1-augustus-gene-256.35-mRNA-1	0.000819148	21
37	16	*F.iinumaeNPR31*	maker-Fvb3-2-augustus-gene-63.42-mRNA-1	0.000819148	21
38	16	*F.niponicaNPR31*	maker-Fvb3-3-augustus-gene-47.46-mRNA-1	0.000819148	21
39	16	*F.viridisNPR31*	maker-Fvb3-1-augustus-gene-256.35-mRNA-1	0.000819148	21
40	20	*F.iinumaeNPR31*	maker-Fvb3-2-augustus-gene-63.42-mRNA-1	0.00997927	18
41	20	*F.niponicaNPR31*	maker-Fvb3-3-augustus-gene-47.46-mRNA-1	0.00997927	18
42	20	*F.viridisNPR31*	maker-Fvb3-1-augustus-gene-256.35-mRNA-1	0.00997927	18

B

№	Sequence (5´-3´)	Start
1	CTCGGATGATGTTGAACTTGTGA	11
2	GAGTCGAATATAACCTTAGATGA	48
3	AGCCTACTGTGATCCTAAGGTTG	95
4	ATGGCTGATGTTAACCTTCGTAA	141
5	TGGCTGATGTTAACCTTCGTAAT	142
6	GGCTGATGTTAACCTTCGTAATC	143
7	GGGTTATACCGTGCTTCATATTG	170
8	TACCGTGCTTCATATTGCTGTGA	176
9	CCGTGCTTCATATTGCTGTGATG	178
10	CGCAGAGAGCCATCAATAATAGT	201
11	GAGAGCCATCAATAATAGTATTG	205
12	GAGCTGACATCAGATGGTCAAAG	255
13	CTGACATCAGATGGTCAAAGTGC	258
14	CAGATGGTCAAAGTGCAGTTAGT	265
15	TTGACAAGGGCAAAGGACTATCA	300
16	GACAAGGGCAAAGGACTATCATT	302
17	GGGCAAAGGACTATCATTCAAAA	307
18	AAGGACCGAATTTGCATTGATGT	354
19	TAGAGAGAGAAATGCGGAAGAAT	379

D

№	siRNA	*Homeologous*	Target Name	E-value	ID
1	1	*F.iinumaeNPR31*	maker-Fvb3-2-augustus-gene-63.42-mRNA-1	6.71529E-5	23
2	1	*F.niponicaNPR31*	maker-Fvb3-3-augustus-gene-47.46-mRNA-1	6.71529E-5	23
3	2	*F.iinumaeNPR31*	maker-Fvb3-2-augustus-gene-63.42-mRNA-1	6.71529E-5	23
4	2	*F.niponicaNPR31*	maker-Fvb3-3-augustus-gene-47.46-mRNA-1	6.71529E-5	23
5	2	*F.viridisNPR31*	maker-Fvb3-1-augustus-gene-256.35-mRNA-1	6.71529E-5	23
6	3	*F.iinumaeNPR31*	maker-Fvb3-2-augustus-gene-63.42-mRNA-1	6.71529E-5	23
7	3	*F.niponicaNPR31*	maker-Fvb3-3-augustus-gene-47.46-mRNA-1	6.71529E-5	23
8	4	*F.niponicaNPR31*	maker-Fvb3-3-augustus-gene-47.46-mRNA-1	6.71529E-5	23
9	4	*F.viridisNPR31*	maker-Fvb3-1-augustus-gene-256.35-mRNA-1	6.71529E-5	23
10	4	*F.iinumaeNPR31*	maker-Fvb3-2-augustus-gene-63.42-mRNA-1	0.00285541	18
11	5	*F.niponicaNPR31*	maker-Fvb3-3-augustus-gene-47.46-mRNA-1	6.71529E-5	23
12	5	*F.viridisNPR31*	maker-Fvb3-1-augustus-gene-256.35-mRNA-1	6.71529E-5	23
13	6	*F.niponicaNPR31*	maker-Fvb3-3-augustus-gene-47.46-mRNA-1	6.71529E-5	23
14	6	*F.viridisNPR31*	maker-Fvb3-1-augustus-gene-256.35-mRNA-1	6.71529E-5	23
15	7	*F.iinumaeNPR31*	maker-Fvb3-2-augustus-gene-63.42-mRNA-1	6.71529E-5	23
16	7	*F.viridisNPR31*	maker-Fvb3-1-augustus-gene-256.35-mRNA-1	6.71529E-5	23
17	8	*F.iinumaeNPR31*	maker-Fvb3-2-augustus-gene-63.42-mRNA-1	6.71529E-5	23
18	8	*F.viridisNPR31*	maker-Fvb3-1-augustus-gene-256.35-mRNA-1	6.71529E-5	23
19	8	*F.niponicaNPR31*	maker-Fvb3-3-augustus-gene-47.46-mRNA-1	0.00285541	19
20	9	*F.iinumaeNPR31*	maker-Fvb3-2-augustus-gene-63.42-mRNA-1	6.71529E-5	23
21	9	*F.viridisNPR31*	maker-Fvb3-1-augustus-gene-256.35-mRNA-1	6.71529E-5	23
22	9	*F.niponicaNPR31*	maker-Fvb3-3-augustus-gene-47.46-mRNA-1	0.00081809	21
23	10	*F.iinumaeNPR31*	maker-Fvb3-2-augustus-gene-63.42-mRNA-1	6.71529E-5	23
24	10	*F.niponicaNPR31*	maker-Fvb3-3-augustus-gene-47.46-mRNA-1	6.71529E-5	23
25	10	*F.viridisNPR31*	maker-Fvb3-1-augustus-gene-256.35-mRNA-1	0.00285541	20
26	11	*F.iinumaeNPR31*	maker-Fvb3-2-augustus-gene-63.42-mRNA-1	6.71529E-5	23
27	11	*F.niponicaNPR31*	maker-Fvb3-3-augustus-gene-47.46-mRNA-1	6.71529E-5	23
28	11	*F.viridisNPR31*	maker-Fvb3-1-augustus-gene-256.35-mRNA-1	6.71529E-5	23
29	12	*F.iinumaeNPR31*	maker-Fvb3-2-augustus-gene-63.42-mRNA-1	6.71529E-5	23
30	12	*F.niponicaNPR31*	maker-Fvb3-3-augustus-gene-47.46-mRNA-1	6.71529E-5	23
31	12	*F.viridisNPR31*	maker-Fvb3-1-augustus-gene-256.35-mRNA-1	6.71529E-5	23
32	13	*F.iinumaeNPR31*	maker-Fvb3-2-augustus-gene-63.42-mRNA-1	6.71529E-5	23
33	13	*F.niponicaNPR31*	maker-Fvb3-3-augustus-gene-47.46-mRNA-1	6.71529E-5	23
34	13	*F.viridisNPR31*	maker-Fvb3-1-augustus-gene-256.35-mRNA-1	6.71529E-5	23
35	14	*F.iinumaeNPR31*	maker-Fvb3-2-augustus-gene-63.42-mRNA-1	6.71529E-5	23
36	14	*F.niponicaNPR31*	maker-Fvb3-3-augustus-gene-47.46-mRNA-1	6.71529E-5	23
37	14	*F.viridisNPR31*	maker-Fvb3-1-augustus-gene-256.35-mRNA-1	6.71529E-5	23
38	17	*F.iinumaeNPR31*	maker-Fvb3-2-augustus-gene-63.42-mRNA-1	0.00285541	20
39	17	*F.niponicaNPR31*	maker-Fvb3-3-augustus-gene-47.46-mRNA-1	0.00285541	20
40	17	*F.viridisNPR31*	maker-Fvb3-1-augustus-gene-256.35-mRNA-1	0.00285541	20
41	18	*F.iinumaeNPR31*	maker-Fvb3-2-augustus-gene-63.42-mRNA-1	6.71529E-5	23
42	18	*F.niponicaNPR31*	maker-Fvb3-3-augustus-gene-47.46-mRNA-1	6.71529E-5	23
43	18	*F.viridisNPR31*	maker-Fvb3-1-augustus-gene-256.35-mRNA-1	6.71529E-5	23
44	19	*F.iinumaeNPR31*	maker-Fvb3-2-augustus-gene-63.42-mRNA-1	0.00285541	18
45	19	*F.niponicaNPR31*	maker-Fvb3-3-augustus-gene-47.46-mRNA-1	0.00285541	18
46	19	*F.viridisNPR31*	maker-Fvb3-1-augustus-gene-256.35-mRNA-1	0.00285541	18

Figure S5: Candidate DNA fragments of the strawberry FaNPR3.1 for dsRNA production (siRNAs). *(A) and (B) indicate the sequence and the position of each predicted siRNA within the 407 bp DNA fragment of the FaNPR3.1 used for the construction of the corresponding intragenic dsRNAi inducing cassettes, and (C) and (D) BLAST of each candidate dsRNA inducing sequence against v1.0.a1 Transcripts database indicating the identity with its corresponding homoeologous in the F. x ananassa genome. Predicted siRNA sequence were obtained by using the InvivoGen siRNA Wizard software 3.1.(A) and siRNA at Whitehead (B). Search parameters of the BLAST search against Fragaria x ananassa Camarosa Genome transcripts database were adjusted to the short sequence (e-value 0,05 and Match/Mismatch Scores 2/-3).*

Figure S6: Alignment of the coding DNA sequence of the strawberry AAT2 genes.

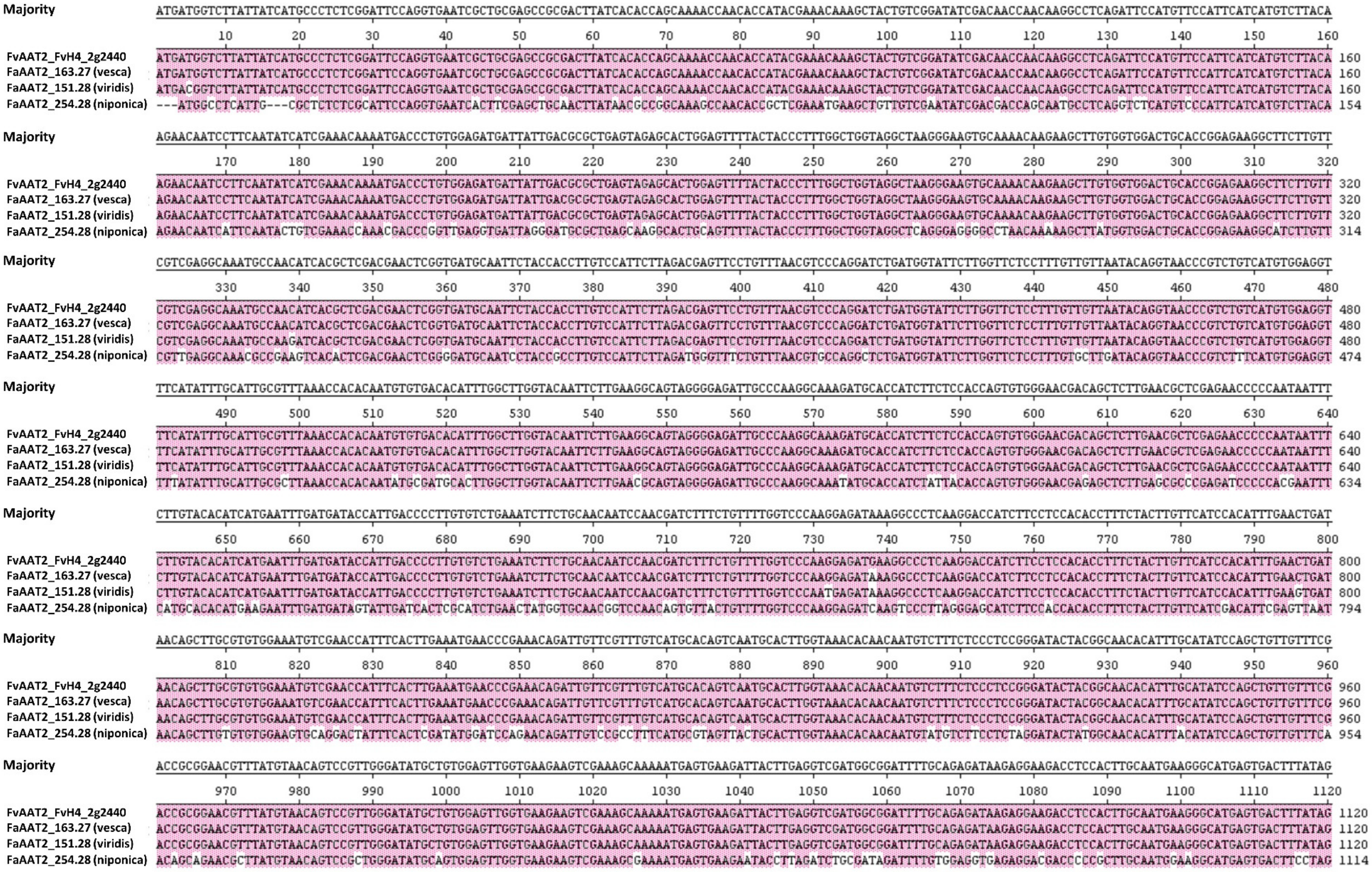

(Cont)

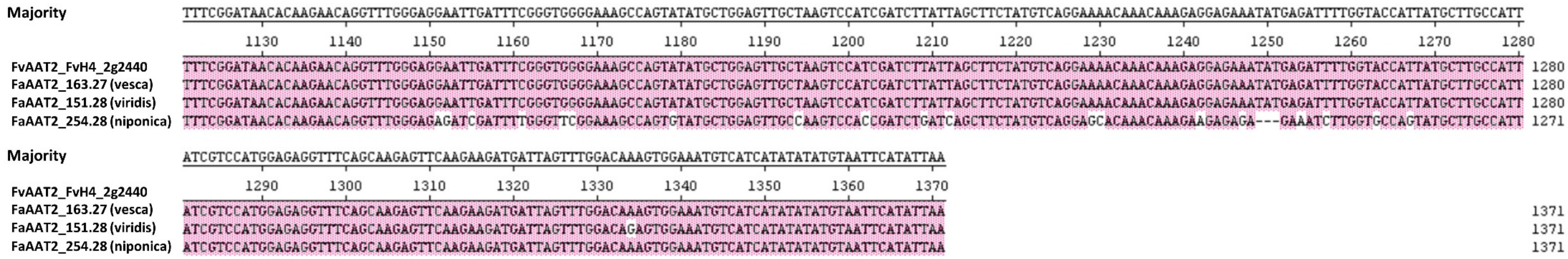

Figure S6: Alignment of the coding DNA sequence of the strawberry AAT2 genes. *Alignment of the 1371 bp DNA fragment corresponding to coding sequence of the FvAAT2 (FvH4_5g24240) with its respective homeologous of F. x ananassa FaAAT2_163.27, maker-Fvb5-1-snap-gene-163.27-mRNA-1 (Vesca homeologous); FaAAT2_151.28, maker-Fvb5-2-snap-gene-151.28-mRNA-1 (Viridis homeologous); and FaAAT2_254.28 maker-Fvb3-3-augustus-gene-254.28-mRNA-1 (niponica homeologous).*

Figure S7: Alignment of the coding DNA sequence of the strawberry DOF2 genes.

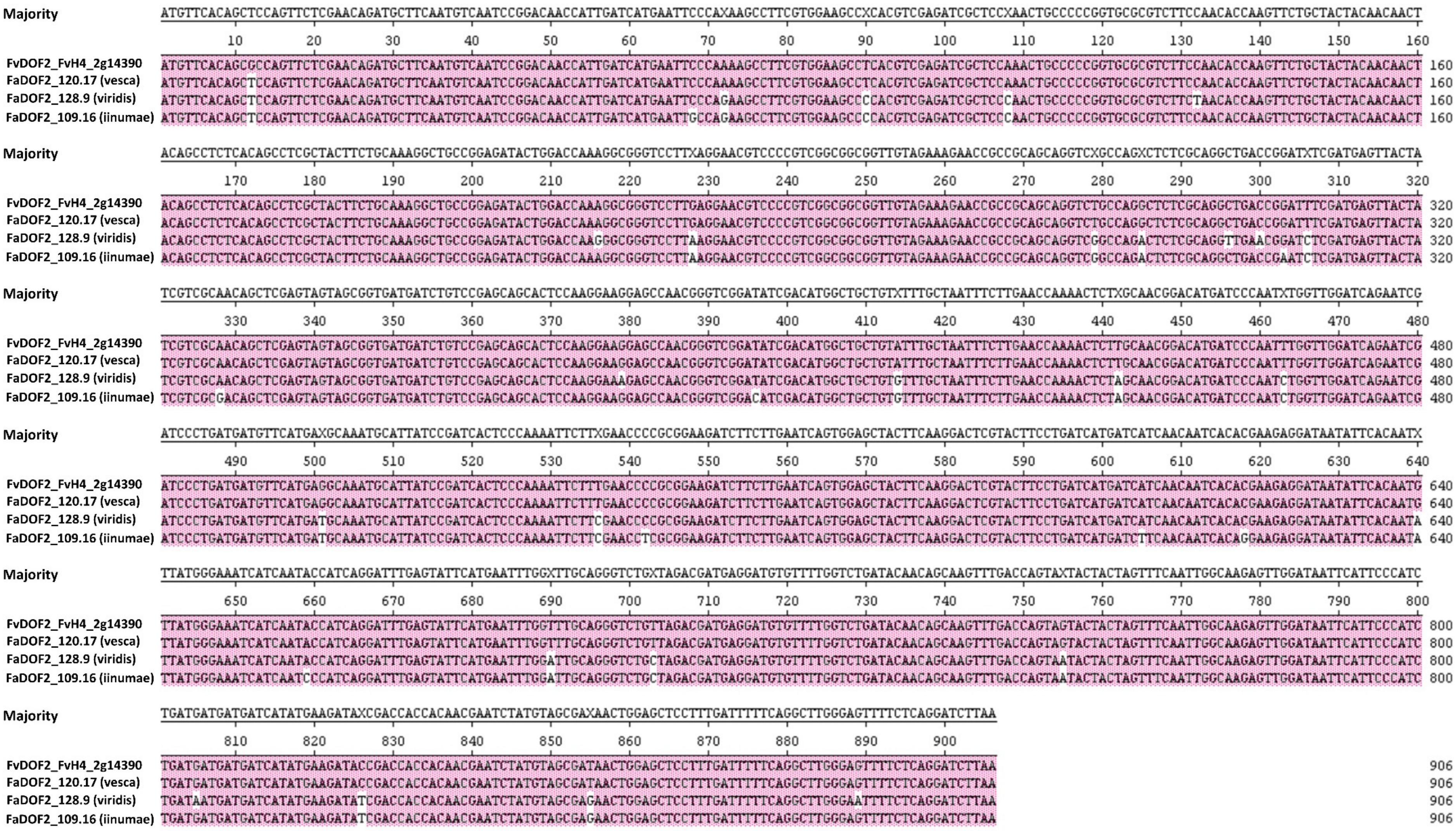

Figure S7*: *Alignment of the coding DNA sequence of the strawberry DOF2 genes. *Alignment of the 906bp DNA fragment corresponding to the coding sequence of FvDOF2 (FvH4_2g14390) with its respective homeologous of F. x ananassa FaDOF2_120.17, snap_masked-Fvb2-2-processed-gene-120.17-mRNA-1 (Vesca homeologous); FaDOF2_128.9, augustus_masked-Fvb2-3-processed-gene-128.9-mRNA-1(Viridis homeologous); and FaDOF2_109.16 snap_masked-Fvb2-4-processed-gene-109.16-mRNA-1 (iinumae homeologous). All sequences were retrieved of Fragaria vesca v.4.0a1 and Fragaria x ananassa Camarosa Genome v1.0.a1 Transcripts database*

FigureS8. Expression analysis of the strawberry FaDOF2 in green and red fruit receptacles of F. vesca and F. × ananassa cv. Camarosa

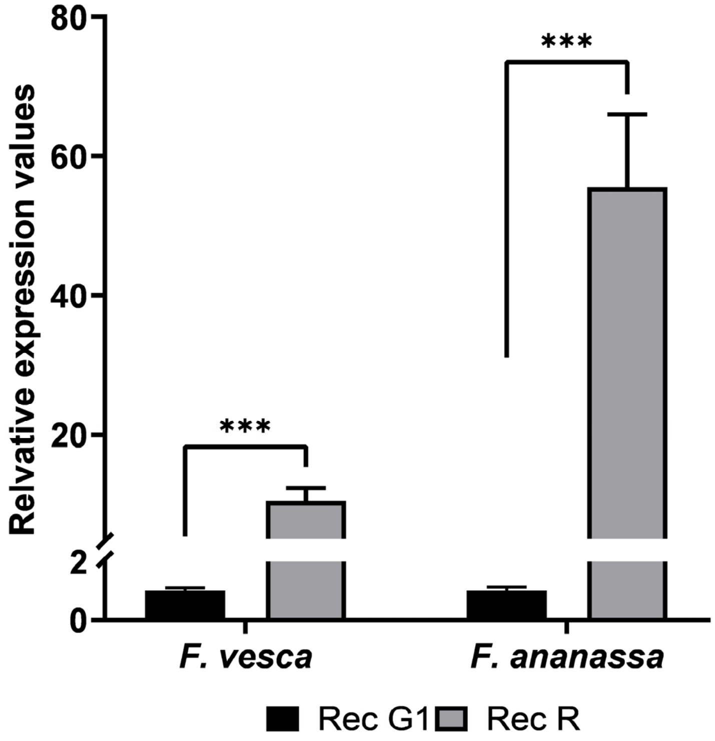

Figure S8. Expression analysis of the strawberry FaDOF2 in green and red fruit receptacles of F. vesca and F. × ananassa cv. Camarosa. *qRT-PCR results were obtained using specific primers for FaDOF2 (Molina-Hidalgo et al., 2017). The increase in the mRNA value was relative to the receptacle G1 in all experiments, which was assigned an arbitrary value equal to 1. Means values ± SD of five independent experiments are shown. Rec G1: small-sized green fruit receptacle; Rec R: red stage fruit receptacle. Statistical significance with respect to reference sample was determined by the Student's t-test in all experiments. (***) p-value < 0.001.*

Table S1.Primers

**Name**	**Description**	**Chain**	**RT-qPCR (5´-3´)**	**Observation**
**NptII_Fw**	NPTII	sense	GACCACCAAGCGAAACATCG	Amplicon of 356 bp of kanamycin cassettes in the pKGWFS7.0 used as housekkeping
**NptII_Rv**		anti-sense	TGCGAATCGGGAGCGGCGATACCG	
**GUS_Fw**	GUS	sense	AATTATGCGGGCAACGTCTGGTAT	Amplicon of 203 bp of Glucuronidase gene in pKGWFS7.0
**GUS_RV**		anti-sense	CGTACACTTTTCCCGGCAATAACA	

Table S1. Primers used in the RT-qPCR in this study
